# Pathogen species and capsule expression shape opportunistic transfer and the recipient response during plasmid RP4 conjugation

**DOI:** 10.1038/s42003-026-10359-w

**Published:** 2026-05-27

**Authors:** Galain C. Williams, Jillian C. Danne, Simon Crawford, Jihane Homman-Ludiye, Xenia Kostoulias, Bailey Heron, Tasnia Islam, Melvin Yong, Yunn-Hwen Gan, Yogitha N. Srikhanta, Dena Lyras

**Affiliations:** 1 Infection Program, Monash Biomedicine Discovery Institute and Department of Microbiology, Monash University, Melbourne, Victoria Australia; 2 Monash Ramaciotti Centre for Cryo-Electron Microscopy, A Node of Microscopy Australia, Monash University, Melbourne, Victoria Australia; 3 Monash Micro Imaging, Monash University, Melbourne, Victoria Australia; 4 Department of Infectious Diseases, The Alfred Hospital and School of Translational Medicine, Monash University, Melbourne, Victoria Australia; 5 Infectious Diseases Translational Research Program, Department of Biochemistry, Yong Loo Lin School of Medicine, National University of Singapore, Singapore, Singapore

**Keywords:** Microbial genetics, Antibiotics, Bacterial evolution, Bacterial genetics

## Abstract

Conjugative plasmids drive bacterial evolution and niche adaptation, yet how their active acquisition reshapes host transcription remains poorly understood. Most studies focus on stable plasmid carriage, overlooking the dynamic transcriptional changes during conjugation itself. Here, we investigate the active conjugation of RP4, a prototypical broad-host range plasmid, to *E. coli*, and *K. pneumoniae* recipients. We observe that an immediate, host- and surface factor-dependent transcriptional response occurs. This includes activation of non-SOS stress pathways, motility, exopolysaccharide production, anaerobic respiration, and metabolic adaptation. These responses do not inhibit conjugation, suggesting they serve to maintain host homeostasis, particularly of envelope encoded functions. Capsule expression prevents these responses and inhibits RP4 activation by physically blocking donor-recipient contact. Unexpectedly, RP4 can bypass the capsular barrier through opportunistic transfer into a sub-population of phenotypically thin-capsulated cells. These findings show how RP4, the hosts species, and surface architecture shape the active conjugation transcriptional landscape, which may have implications for plasmid dissemination and bacterial evolution.

## Abstract (web summary)

An intersection between recipient species, and the cell surface shapes the success and transcriptional landscape of plasmid RP4 conjugation; opportunistic transfer of RP4 involves thin-capsulated recipient sub-populations.

## Introduction

Conjugative plasmids are key drivers of horizontal gene transfer, enabling rapid trait acquisition in bacteria^1^. Critically, their spread through bacterial populations is closely linked to the rise of antibiotic resistance across clinical and environmental settings^2,3^. Conjugation, the primary mechanism of plasmid dissemination, involves the transfer of single-stranded plasmid DNA from a donor into a neighbouring recipient *via* a type IV secretion system^4^. Although traditionally viewed as a donor-driven process, recent seminal studies have revealed that recipients play active, multifaceted roles in modulating conjugation^5–8^. For example, the capsule of *Klebsiella pneumoniae* inhibits transfer of many plasmid types^8,9^, whilst *K. pneumoniae* outer membrane proteins act as receptors that selectively engage cognate plasmid-bearing donors^7^. Additionally, plasmid carriage induces widespread changes in recipient chromosomal gene expression^10^, altering cellular functions beyond the plasmid’s genetic content. These findings highlight a complex interplay between donor, recipient and plasmid that shapes plasmid dissemination and influences host cell physiology and evolution. In this study, we aimed to further dissect this interplay during active conjugation of the broad-host range plasmid RP4 in two species, *Escherichia coli* and *K. pneumoniae*, two species with clinical, agricultural, and environmental significance.

Stable plasmid carriage is known to broadly reprogramme host chromosomal gene expression, affecting metabolic, respiratory, transport, virulence, and other pathways^10–14^. However, the transcriptional response of recipient cells during active conjugation, particularly involving broad-host-range plasmids like RP4, remains poorly understood. While many plasmids trigger an SOS response upon entry^15^, RP4 does not strongly activate this pathway^15^, suggesting alternative mechanisms may be at play. Despite RP4’s widespread use in inter-species gene transfer, its transcriptional impact across diverse species is surprisingly underexplored. For example, RP4 has been shown to supress ammonia oxidation in environmental bacteria^16^, but most studies have been conducted in the presence of external compounds such artificial sweeteners^17^, or biochar^18^, confounding the effects of RP4 acquisition with those of the compounds themselves.

Research on other broad-host range plasmids suggests that initial plasmid-host interactions can shape the outcome of transfer. In *Pseudomonas putida*, for instance, R388 transfer induces expression of the *hsdRMS* restriction system, which is counteracted by the R388 anti-restriction protein ArdC^19^. Whether RP4 elicits a similar recipient-specific response or encounters barriers to conjugation remains unknown. Understanding how recipient species modulate RP4 conjugation is critical, as broad-host-range plasmids are major vectors in the spread of antibiotic resistance^20^.

Beyond transcriptional responses, recipient bacteria also influence conjugation through their cell surface structures. Capsules, thick outer layers of polysaccharide, are well-established barriers to conjugation^8^. Paradoxically, they are also associated with increased rates of horizontal gene transfer^21^, suggesting a complex and unresolved relationship between capsule presence and gene exchange. This paradox remains poorly understood, in part because capsule-mediated inhibition of conjugation has only been demonstrated in *Klebsiella* species, and the underlying mechanisms are yet to be elucidated.

Using a liquid displacement method to infer colony volume^8^, Haudiquet et al. showed that larger colony volumes more greatly inhibited plasmid transfer, suggesting but not showing that capsule length was a key factor. Given that cell-to-cell contact is essential for RP4 conjugation^22^, a plausible, though unproven, hypothesis is that capsules physically obstruct donor-recipient contact, thereby preventing conjugation. To deepen our understanding of how recipient bacteria respond to and modulate broad-host range plasmid conjugation, we investigated the transcriptional and structural impact of active RP4 transfer in *E. coli* and *K. pneumoniae*. Both species exhibited distinct transcriptional responses involving diverse host functions, including anaerobic respiration, non-SOS stress adaptation, and substrate transport. Notably, the *K. pneumoniae* response was only observed in the absence of capsule, revealing a previously unrecognised role for the capsule in suppressing host transcriptional reprograming during conjugation.

The expression of RP4-encoded genes was also influenced by the recipient species and was inhibited by the presence of capsule. We confirmed that capsule blocks conjugation across diverse recipients by preventing donor-recipient cell-to-cell contact. Surprisingly, RP4 could opportunistically transfer into genetically thick-capsulated recipients due to single cell variation in capsular thickness, bypassing the need for a serotype switch. These findings provide new insight into how recipient species and their surface structures shape the transcriptional landscape and dissemination of broad-host range plasmids such as RP4.

## Results

### Active RP4 conjugation elicits triggers non-SOS stress, respiratory and metabolic reprogramming

**Fig. 1:**
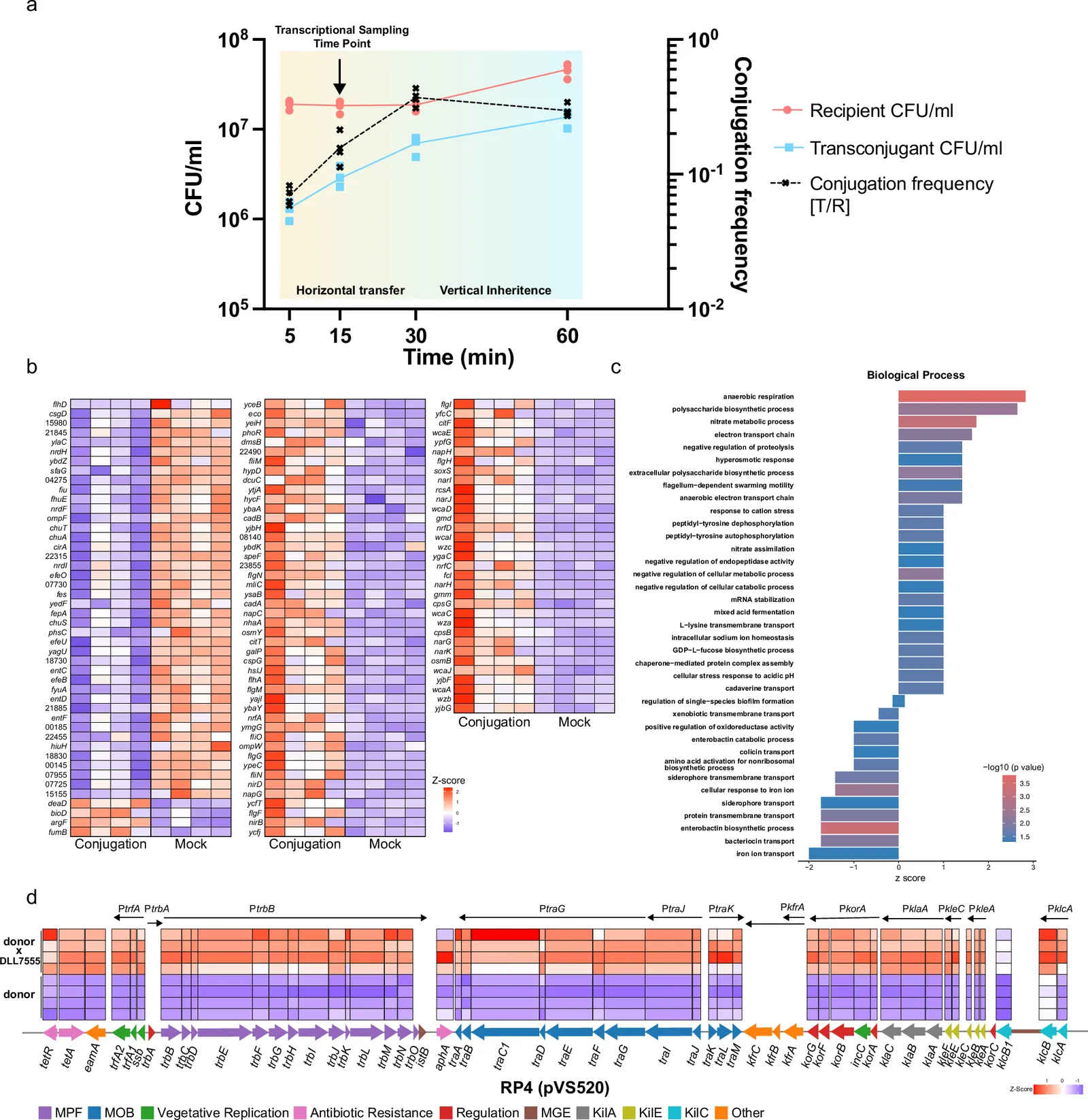
The transcriptional response of an *E. coli* recipient and plasmid RP4 during active conjugation. **a** *E. coli* DLL7555 conjugation frequency over time shows that RP4 conjugation is active at 15 min and completed by 30 min. Arrow indicates the time for transcriptional sampling. **b** Differentially expressed DLL7555 genes during active RP4 conjugation. **c** GO-term analysis of enriched genes with the R TopGo package with the weight_01 algorithm. **d** Differential expression of RP4-encoded genes during active conjugation with *E. coli* DLL7555. Numbers in *E. coli* gene list represent locus tags trimmed of the prefix ACPEGL. Trend line = mean, *N* = 4.

To map the transcriptional landscape of RP4 conjugation with *E. coli* and *K. pneumoniae*, we first distinguished active conjugation from transconjugant vertical inheritance using a time-course assay (Fig. 1a). At 15 min, new transconjugants were actively generated without changes in recipient population size, confirming this as a window of active conjugation and ruling out vertical inheritance as a confounding factor. We then performed RNA-seq on a conjugating mixture of laboratory donor *E. coli* LT101(pVS520) and a genetically distant clinical recipient, *E. coli* DLL7555. For comparison, RNA was also extracted from mock conjugations lacking pVS520 and from donor monocultures. The >78,000 core genome differences between DLL7555 and LT101 enabled accurate, strain-specific read assignment using a concatenated LT101-DLL7555 reference genome.

RP4 conjugation triggered differential expression of 130 genes in the *E. coli* recipient (Fig. 1b), including those involved in adaptation to external stressors such as temperature shock (*cspG*, *hslJ, deaD*), pH regulation (*yagU, ygaC, ybay, speF*), osmotic stress (*osmB, osmY, nhaA*), and other stress-related pathways (*mliC, soxS, rcsA, phoR*). This was accompanied by upregulation of genes involved in colanic acid biosynthesis (*wzc, wzb, cpsB, wcaJ, wcaI, wcaE, wcaC, wcaA, gmd, fcl, wza, wcaD*), exopolysaccharide production (*yjbG, yjbF, yjbH*), and flagella assembly (*fliN, fliM, flgG, flgF, glgI, flgH, flhA, fliO*).

Although conjugative plasmids are known to promote biofilm formation^23^, we observed decreased expression of the biofilm master regulator *csgD*. Consistently, co-culture of DLL7555 with LT101(pVS520) resulted in a slight but significant reduction in biofilm formation compared to both LT101 co-culture or monoculture conditions (Supplementary Fig. 1).

RP4 conjugation also induced upregulation of genes involved in anaerobic respiration and energy generation (*fdnI, dmsB, napC, narG, narH, narI, hycF, nrfA, nrfD, nrfC, nirB, fumB, dcuC*), overlapping with pathways linked to inorganic nutrient metabolism and nitrogen cycling (*napG, napH, narG, narH, narI, nrfA, narJ, nirB, nirD, narK, napC*). In contrast, genes associated with siderophore, iron transport, and iron homeostasis were downregulated, including *fiu, cirA, fepA, fyuA, efeO, efeU, efeB, fhuE, entD, entC, entF, chuA, chuS, chuT*.

Gene Ontology (GO) term analysis reflected this multifaceted recipient response, with significant enrichment in categories related to stress adaptation, respiratory activity, and metabolic reprogramming (Fig. 1c).

By comparing the conjugation condition to the donor alone, we aimed to uncover the global expression profile of RP4 across the entire conjugating population, recognizing that this expression could originate from either donor or transconjugant cells. This analysis revealed a striking upregulation of 47 genes encoded by pVS520, spanning key functional categories including DNA mobilisation (MOB), mating pair formation (MPF), regulation and replication, host-kill systems (*kil*), and other cellular processes (Fig. 1d). These findings confirm prior transcriptional studies of RP4 during *E. coli* conjugation^24^, demonstrating that RP4 undergoes intense transcriptional activation during conjugation with this species, with nearly all transcriptional units switched on.

### Capsule shields the recipient from transcriptional reprogramming during conjugation

To assess whether a similar recipient response to RP4 conjugation also occurred in a separate species, we repeated our experiment using *K. pneumoniae* B5055 as the recipient. This strain’s hypermucoid capsule also allowed us to investigate how recipient cell surface structures influence the transcriptional landscape during conjugation. Capsules are known barriers to conjugation^8^, and prior to RNA-seq, we confirmed that the capsule of *K. pneumoniae* B5055 significantly inhibited RP4 conjugation (supplementary Fig. 2). Since this effect has only been demonstrated in *Klebsiella spp* in vitro, we extended our analysis to *Acinetobacter baumannii* AB5075 and *Pasteurella multocida* VP161, confirming that capsule similarly impedes conjugation in these species (Supplementary Fig. 2). This represents the first in vitro evidence that capsules broadly inhibit conjugation across multiple genera beyond *Klebsiella*.

**Fig. 2:**
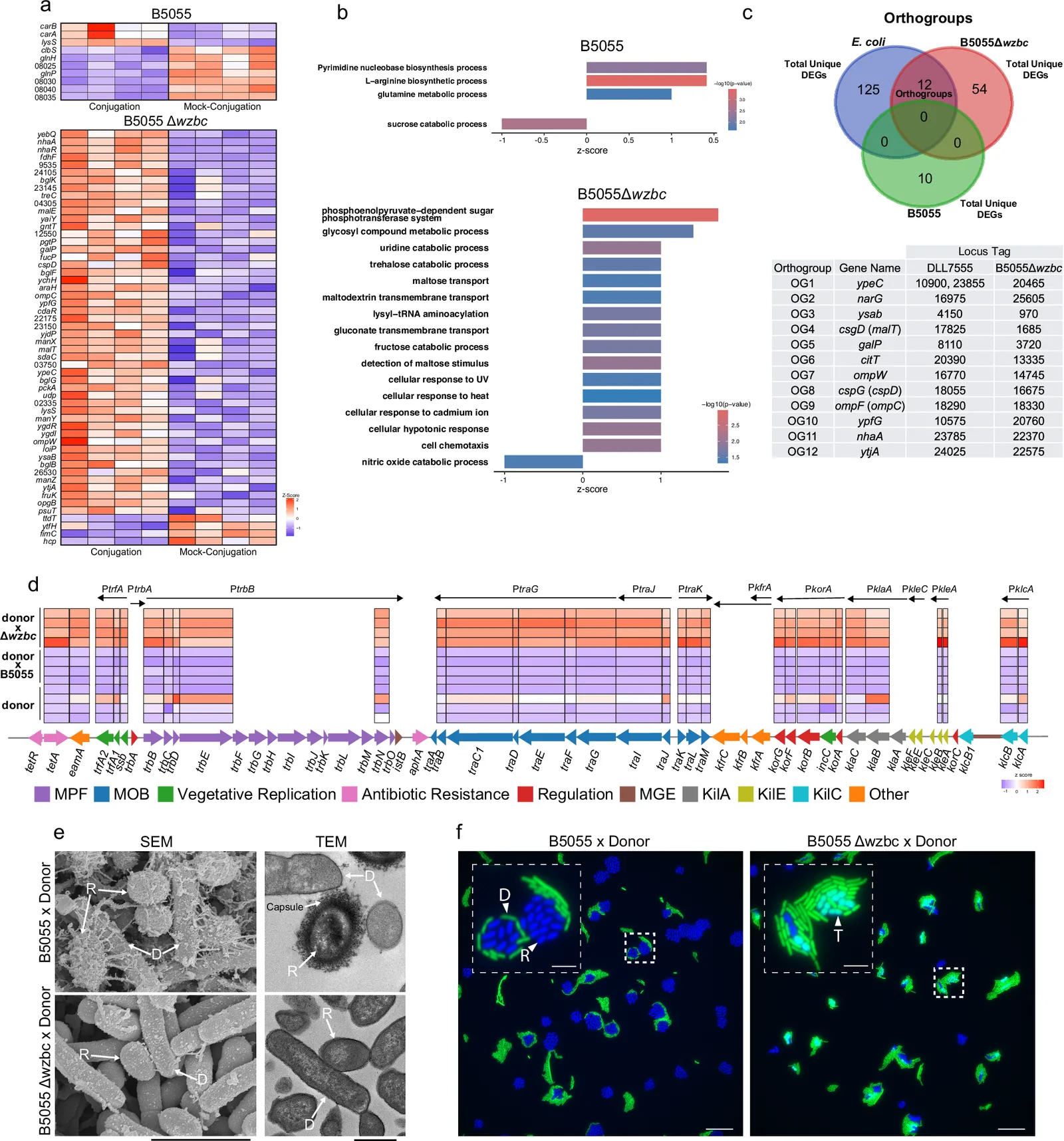
The effect and mechanism of capsule on conjugation and the recipient and plasmid response. **a** Differentially expressed genes (DEGS) in *K. pneumoniae* B5055 (top) and B5055Δ*wzbc* (bottom) genes during active conjugation or co-culture with an RP4 donor. **b** GO-term analysis of DEG for B5055 (top) and B5055Δ*wzbc*, with raw *P* values reported as per TopGO package guidelines for the weight_01 algorithm. **c** Orthogroups that are shared between recipient responses. Brackets indicate the name of the gene in B5055. **d** Differentially expressed RP4 genes during active conjugation with B5055Δ*wzbc* (top), B5055 (middle), or the donor alone (bottom). **e** TEM and SEM images of conjugation between *K. pneumoniae* B5055 or B5055Δ*wzbc* recipients with *E. coli* LT101. Recipients are distinguished based on the presence of a capsule and shape. **f** In situ visualisation of conjugation between either wild-type or Δ*wzbc* recipients by fluorescent microscopy. All images are representative of ≥2 independent experiments. Abbreviations: D = Donor, R = Recipient, T = Transconjugant. Numbers in *K. pneumoniae* and *E. coli* gene/orthologue lists represent locus tags trimmed of the prefix J6T75_RS and ACPEGL, respectively. Scale bars: SEM 3 µm, TEM 1 µm, Fluorescence microscopy 20 µm, inset 5 µm.

To investigate how the capsule influences the transcriptional response to conjugation, we compared gene expression profiles in wild-type and acapsular *K. pneumoniae* recipients. In the wild-type strain, only 10 genes were differentially expressed (including *carB, carA, lysS, clbS, glnH*, 08025*, glnP*, and several uncharacterized loci: 08030, 08050, 08045*)* (Fig. 2a). These genes were associated with pathways such as sucrose catabolism, L-arginine biosynthesis and nucleotide biosynthesis (Fig. 2b).

However, when the acapsular mutant B5055Δ*wzbc* was used as the recipient, the transcriptional response expanded dramatically to 54 genes. This striking increase demonstrates that the capsule not only acts as a physical barrier to conjugation but also shields the recipient from widespread transcriptional reprogramming. By limiting conjugation frequency, the capsule effectively insulates the host transcriptome from the impact of plasmid acquisition.

Building on this observation, we further examined this transcriptional landscape in the absence of capsule. *K. pneumoniae* B5055Δ*wzbc* exhibited upregulation of stress adaptation genes shared with *E. coli* (e.g. *cspD*, *nhaA*), as well as responses unique to this strain (*loiP*, *ychH, hcp*). We also observed broad changes in genes involved in carbohydrate transport (*araH, fucP, galP, manY, manZ, manX, bglF*), catabolism (*treC, fruK, pck, bglB, opbG*), and transmembrane transport (*psuT, sdaC, ompC, ttdT, nhaA, yebQ, malE, gntT, ompW*).

Consistent with the *E. coli* response, genes linked to anaerobic growth (*ompW*, *ttdT*) and anaerobic respiration (*fdnG, fdhF, narG*) were also activated. GO-term analysis highlighted enrichment in mixed stress responses and altered carbohydrate transport and metabolism (Fig. 2b). Using Orthofinder, we identified 12 shared orthogroups that were differentially expressed during active conjugation in both *E. coli* DLL7555 and *K. pneumoniae* B5055Δ*wzbc* (Fig. 2c). These conserved responses included (*ypeC*, *narG*, *ysaB*, *csgD*, *malT*, *galP*, *citT*, *ompW*, *cspG*, *cspD*, *ompF*, *ompC*, *ypfG*, *nhaA*, and *ytjA*), indicating that approximately 10% of the transcriptional response to RP4 is conserved.

### Capsules block conjugation by preventing donor cell activation and cell-to-cell contact

Capsules are potent inhibitors of bacterial conjugation, widely believed to block essential donor-recipient contact, yet this mechanism has not been demonstrated. Before examining their role in physical contact, we first asked whether capsules influence the activation of RP4-encoded genes during conjugation. To address this, we re-analysed our transcriptomic dataset comparing wild-type *K. pneumoniae* and its acapsular mutant B5055Δ*wzbc*. When the recipient was encapsulated, no RP4 genes were activated (Fig. 2d). In contrast, conjugation with the acapsular mutant triggered expression of 27 genes. Notably, this was fewer than the number activated during conjugation with *E. coli*, suggesting that RP4 gene expression is shaped by the specific recipient species.

To determine whether RP4 gene activation originated from the donor or transconjugant cells, we employed a donor-specific fluorescent reporter assay targeting the relaxase promoter. By using rifampicin to selectively silence recipient transcription, taking advantage of donor rifampicin resistance, we confirmed that relaxase operon activation occurred exclusively in donor cells. Crucially, this activation was blocked when the recipient was encapsulated (Supplementary Fig. 3).

These findings reveal that the capsule prevents the donor from sensing the recipient, thereby suppressing transcriptional activation of RP4. This highlights a previously unrecognized role of the capsule in modulating donor behaviour during conjugation.

**Fig. 3:**
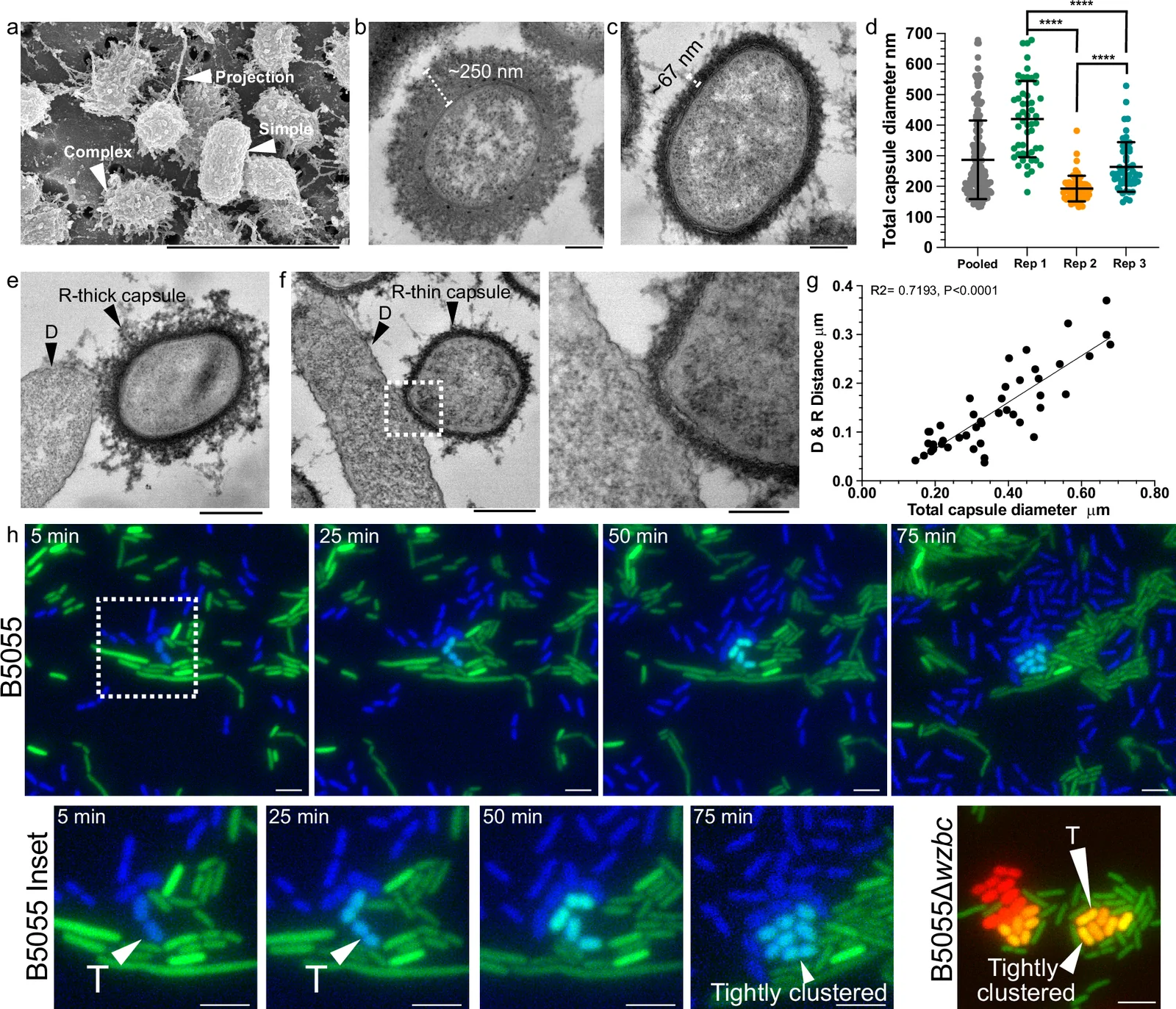
Recipient *K. pneumoniae* capsule thickness variation impacts donor-recipient interactions and conjugation. **a** SEM imaging of capsular variation and projections. **b**, **c** TEM imaging of capsule thickness variation between individual cells. **d** Variation in capsule thickness within, and between biological replicates (*n* = ≥50 cells per sample, Kruskal-Wallis one-way ANOVA with Dunn’s multiple comparison testing *P* values = **** ≤ 0.0001.). **e**, **f** Effect of capsule thickness variation on donor-recipient interactions. **g** Linear regression analysis of distance between outer membrane of donor and recipient and total capsular diameter (*R*^2^ = 0.72, *N* = 47, *F*-test *P* ≤ 0.0001). **h** Live-cell imaging of B5055 transconjugant formation, showing that B5055 transconjugants arise from recipients intimately contacting the donor and morphologically resemble acapsular B5055Δ*wzbc*. Error bars = mean ± SD. Scale bars: a = 5 µm, bc = 200 nm, ef = 500 nm, f inset = 200 nm, h = 20 µm, h insert = 5 µm. Images are representative of ≥2 independent experiments, except for (**h**), which depicts the rare formation of a B5055 transconjugant.

To uncover the mechanism by which capsule inhibits conjugation, we performed imaging studies to directly visualize donor-recipient interactions in the presence and absence of capsule. Transmission and scanning electron microscopy revealed that the capsule of *K. pneumoniae* physically obstructs direct outer membrane contact between donor and recipient cells (Fig. 2e, top panels). In contrast, when the capsule was absent, intimate membrane-to-membrane contact was readily observed (Fig. 2e, bottom panels), consistent with conditions permissive for conjugation. Unexpectedly, these imaging studies also revealed substantial heterogeneity in capsule thickness and architecture at the single-cell level, a phenomenon explored further in a subsequent section (See Fig. 3).

To directly test whether capsule-mediated inhibition of cell-to-cell contact blocks conjugation, we performed fluorescence microscopy on agarose pad conjugation assays (Fig. 2f). We used an RP4-mobilisable plasmid encoding GFP to track plasmid transfer into recipient cells labelled with BFP on a non-mobilizable plasmid. In conjugations with acapsular mutant B5055Δ*wzbc*, 70% of donor-contacting recipient cells successfully acquired the plasmid (369 transconjugants from 527 mating pairs; Fig. 2f, right panel). In stark contrast, only 0.58% of donor-contacting wild-type recipient cells formed transconjugants (2 of 345 mating pairs), and overall mating pair formation was reduced due to capsule-mediated inhibition of direct contact (Fig. 2f, left panel). Notably, wild-type *K. pneumoniae* B5055 cells also showed limited contact with each other, indicating that the capsule restricts both intracellular and intercellular interactions.

Together, these results demonstrate that the capsule prevents donor recognition of the recipient by physically blocking cell-to-cell contact, thereby suppressing conjugation.

### RP4 can bypass the capsule conjugation barrier through opportunistic transfer into thin-capsulated recipient sub-populations

During earlier imaging experiments, we observed striking variability in *K. pneumoniae* capsular thickness at the single-cell level, prompting a more detailed analysis using SEM and TEM (Fig. 3).

SEM revealed that the capsule forms a complex cell surface matrix, often connecting adjacent cells *via* thick, filamentous projections ranging from 0.5 µm to 4 µm in length. These structures were present in 86% of cells examined (581/676), while a minority displayed smooth, unstructured surfaces (Fig. 3a).

TEM further showed that the capsule appears as a diffuse, often non-uniform layer, with substantial variation in thickness between individual cells (Fig. 3b, c). Quantification of total capsule diameter across biological replicates revealed a wide range, from 132.8 nm to 678.8 nm, with a mean of 287 nm (Fig. 3d). Surprisingly, even under identical growth and processing conditions, capsule thickness varied significantly between replicates, with mean values of 420.2 nm, 192.7 nm, and 263.3 nm, respectively (Fig. 3d).

These findings demonstrate that within clonal populations *K. pneumoniae* can exhibits pronounced phenotypic heterogeneity in capsule architecture, highlighting a potential vulnerability that may serve as a bypass to capsule-mediated conjugation barriers.

To determine whether variability in recipient capsule thickness enables rare conjugation events in wild-type *K. pneumoniae*, we investigated whether thinner-capsule cells were responsible for the small number of transconjugants observed. Since capsule blocks conjugation by preventing close donor-recipient contact (Fig. 2g, h), we hypothesized that cells with thinner capsules might permit tighter membrane interactions, allowing plasmid transfer to occur. To test this, we conducted further experiments to directly link capsule heterogeneity with conjugation efficiency.

Supporting this hypothesis, both TEM and SEM imaging revealed that B5055 recipient cells with thinner capsules formed significantly closer associations with donor cells compared to those with thicker capsules (Fig. 3e, f). Quantitative analysis confirmed a strong correlation between capsule thickness and the physical distance separating donor and recipient outer membranes (Fig. 3g, *R*^2^ = 0.72, *N* = 47, *F* test *P* < 0.0001), indicating that thicker capsules physically increase intercellular spacing and reduce the likelihood of conjugation.

Crucially, live-cell fluorescence microscopy showed that transconjugants arose specifically from recipient cells capable of forming intimate contact with donors (Fig. 3h). These transconjugant cells appeared in tightly packed clusters and displayed a morphology strikingly similar to the acapsular B5055Δ*wzbc* mutants (Fig. 3h, inset), consistent with them being thin-capsulated.

Together, these findings demonstrate that natural heterogeneity in capsule thickness enables a subpopulation of recipient cells to bypass the capsule barrier, facilitating rare but successful conjugation events.

**Fig. 4:**
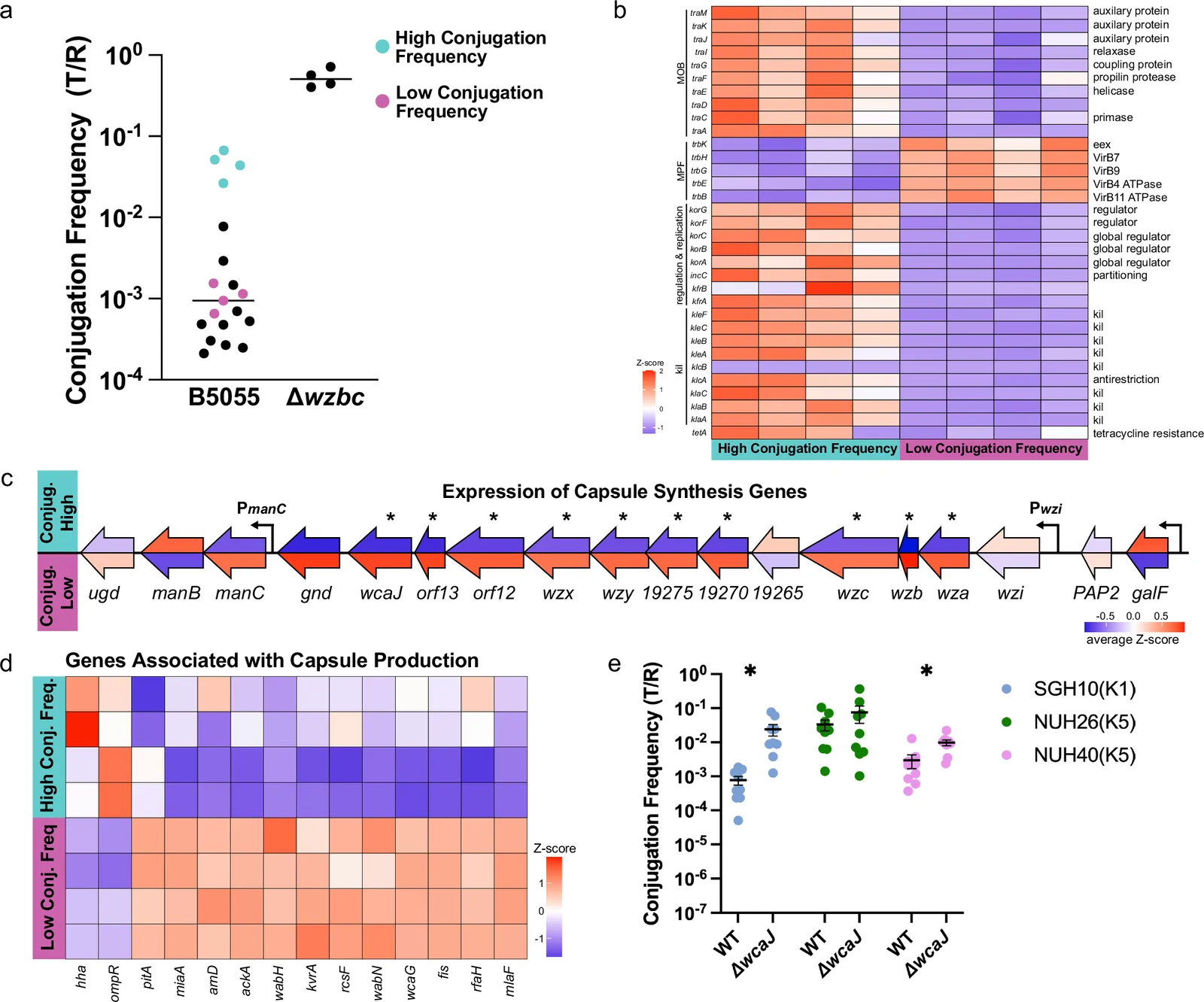
Population level capsule variation enables RP4 conjugation in capsulated *K. pneumoniae.* **a** Variation in conjugation frequency of *K. pneumoniae* B5055. The conjugation frequency of B5055Δ*wzbc* is shown for context. **b** Differential expression of RP4 encoded genes between conjugation high and low samples. **c** Differential expression of genes in the capsule synthesis locus between conjugation high (top) and low (bottom) samples. **d** Genes associated with capsule production between samples with high or low conjugation frequencies. **e** The conjugation frequency of RP4 to hypermucoid K1 or K5 *K. pneumoniae* (*N* = 9, *P* > 0.05 Mann-Whitney *U* test). Data are presented as the median (**a**), or the mean + SEM (**e**).

Given the observed variability in capsule thickness across identically treated replicates (Fig. 3d), we hypothesized that this heterogeneity could occasionally permit higher conjugation frequencies in the wild-type B5055 *K. pneumoniae* recipient. To test this, we conducted 19 independent conjugation experiments, anticipating that natural fluctuations in capsule thickness between replicates would influence conjugation efficiency. In 4 of these experiments, we observed a markedly elevated mean transfer frequency of 4.2 × 10^−2^ (Fig. 4A), approximately ~50-fold higher than the median across all replicates, yet still 20-fold lower than that observed with the acapsular B5055Δ*wzbc* mutant (Fig. 4a). This suggests that while the capsule was still present, its barrier function was partially compromised. Importantly, the resulting transconjugants remained mucoid and string-test positive, confirming they were not spontaneous capsule-deficient mutants.

These findings indicate that variation in capsule expression can transiently weaken its barrier function, enabling rare but enhanced conjugation events in otherwise encapsulated populations.

To investigate the molecular basis of elevated conjugation in wild-type *K. pneumoniae* replicates, we performed RNA-seq to compare RP4 conjugation events and recipient capsule biosynthesis activity between high-frequency and low-frequency conjugation events. In high-frequency conjugations, RP4 gene expression resembled that observed in the acapsular B5055Δ*wzbc* mutant (See Fig. 2d), characterized by strong upregulation of DNA mobilization genes, but not mating pair formation genes (Fig. 4b).

Strikingly, these samples also showed markedly reduced expression of key capsule biosynthesis genes under the control of the P*wzi* promoter (Fig. 4c). Beyond *wzi*,13 additional capsule-associated genes^25^ were differentially expressed, including the master capsule synthesis regulator *kvrA*^26^, and a suite of regulatory and structural genes, such as *hha, ompR, pitA, miaA, arnD, ackA, wabH, rcsF, wabN, wcaG, fis, rfaH* and *mlaF* (Fig. 4d). Many other genes with diverse function were also differentially expressed, suggesting a broader physiological shift in these high-frequency conjugation conditions.

Together, these data reveal that reduced capsule gene expression underlies the increased conjugation efficiency in certain wild-type replicates, reinforcing the role of capsule as a dynamic and regulatable barrier to horizontal gene transfer.

Finally, to assess how variation in capsule serotype influences conjugation, we examined the impact of different hypermucoid capsule types on RP4 transfer efficiency. (Fig. 4e) Conjugation with a hypermucoid K1 recipient resulted in a dramatic 31-fold reduction in transfer frequency compared to its acapsular counterpart. In contrast, two hypermucoid K5 recipients showed more modest reductions of 3.3 and 2.2-fold, relative to their respective wild-type controls. These findings demonstrate that while capsule consistently acts as a barrier to conjugation, the magnitude of its inhibitory effect varies by serotype and is independent of if the serotype is hypermucoid. This highlights that both dynamic capsular thickness and serotype-specific properties govern conjugation efficiency through mechanisms that are partially independent of capsular serotype.

## Discussion

We investigated how recipient *E. coli* and *K. pneumoniae*, capsule shapes, the early transcriptional response and ultimate success of plasmid RP4 conjugation. Our transcriptomic analyses revealed that in the species tested, plasmid acquisition is a highly transcriptionally active process, triggering non-SOS stress responses and metabolic reprogramming in recipient cells. Notably, we found that the bacterial capsule acts as a protective barrier, shielding recipients from this transcriptional upheaval by physically blocking donor-recipient contact and thereby preventing donor activation. We further demonstrated that this inhibitory effect is not unique to *K*. *pneumoniae* since capsules from *A. baumannii and P. multocida* also suppressed conjugation, establishing for the first time capsules as broad-spectrum barriers to conjugation. Strikingly, we discovered that the capsule is not an absolute barrier. Natural heterogeneity in capsule thickness at the single-cell level can allow RP4 to bypass this defence, enabling sporadic but successful plasmid transfer into otherwise resistant populations. Together, our findings provide a new understanding of how recipient bacteria respond to and modulate the transcriptional landscape and efficiency of RP4 conjugation. This work offers new insights into how broad-host range plasmids overcome physical barriers to disseminate across diverse bacterial species.

A key finding of this study is that RP4 conjugation induced an extensive transcriptional response in *E. coli* and *K. pneumoniae* that was previously unknown, extending well beyond the canonical SOS response^27^. Across both *E. coli* and *K. pneumoniae*, conjugation triggered broad non-SOS stress responses and widespread reprogramming of respiratory, metabolic, substrate transport, and other pathways. Many of these pathways operate within the cellular envelope, and the expression of diverse envelope stress markers in both species, such as *osmB, nhaA, hslJ* and *loiP,* indicates that active RP4 acquisition perturbs envelope function and integrity of *E. coli* and *K. pneumoniae*. This was most pronounced in *E. coli* via activation of the auxiliary arm of the Rcs envelope stress regulon^28^, characterised by upregulation of the transcriptional regulator *rcsA* and induction of the colanic acid biosynthesis operon. Colanic acid secretion is stimulated by physical or environmental stressors that threaten membrane integrity, such as osmotic shock^29^. Its expression stabilises envelope integrity, and the proton motive force^30^, and our observation that it is upregulated during conjugation is strongly indicative of recipient envelope shock.

Consistent with an envelope stress response, both species exhibited a distinct anaerobic respiratory shift, marked by the induction of genes such as *ompW, fdnI, narG*, along with indicators of oxidative stress, including *soxS, hcp* and *ychH*. In *E. coli*, envelope stress is associated with decreased aerobic respiration and a transition to anaerobic respiratory pathways^31,32^. The transition during conjugation may be reflective of perturbed envelope integrity but could also reflect increased energy demand associated with the response to conjugation and plasmid acquisition.

Beyond envelope stress, the transcriptional response to RP4 conjugation also revealed considerable crosstalk between diverse biological processes within the recipients tested. In *E. coli*, this was characterised by a strong upregulation of flagellar assembly and exopolysaccharide genes alongside decreased expression of the biofilm master regulator *csgD*. A reduction in biofilm formation was unexpected, as prior reports have suggested RP4 induces biofilm formation in laboratory *E. coli*^23^. However, our functional assays confirmed that active conjugation resulted in a measurable decrease in biofilm formation under the conditions tested. Two differences may explain these differing observations. Firstly, Ghigo et al. studied isolated transconjugants, whilst we studied conjugating mixtures, which reflects the conditions under which *csgD* expression was decreased. Secondly, when considering the increased expression of motility pathways, we hypothesise that this indicates a strain-specific “flight response” where the recipient cell increases motility to physically distance itself from the donor, thereby delaying or preventing the transition to a sessile biofilm lifestyle. This model may explain the discrepancy with earlier work; many common laboratory *E. coli* strains are poorly or non-motile^33^ and would thus be unable to mount such a response, potentially leading to the divergent biofilm phenotypes reported previously. In *K. pneumoniae* motility signals were not observed, as this species is naturally non-motile and lacks flagellar machinery.

Approximately 10% of the recipient transcriptional response was shared between *E. coli* and *K. pneumoniae*. Notably, two conserved genes, *ompW* and *nhaA*, have previously been shown to influence the conjugation efficiency of multiple plasmids, including R388 and F-like plasmids^7,34^. OmpW, typically expressed during the transition to anaerobic respiration^35^, is targeted by mating pair stabilization proteins during conjugation^7^. NhaA, a key regulator of sodium ion, pH, and osmotic balance^36^, also contributes to cell envelope integrity^37^, suggesting a protective role against conjugation-induced envelope stress.

Importantly, this transcriptional upheaval was largely absent in wild-type *K*. *pneumoniae*, where capsule production effectively suppressed the recipient response. Although only a small number of genes were differentially expressed in the encapsulated strain, GO-term analysis still identified numerous redundant terms, a known limitation of this method^38^. In contrast, the broader and more diverse gene expression changes observed in *E. coli* and the acapsular mutant yielded a wide array of non-redundant GO terms, reinforcing the conclusion that capsule acts as a transcriptional shield during conjugation.

Capsules have long been implicated as barriers to conjugation^9,39^, but here we provide the first direct visual evidence that they physically block the essential donor-recipient contact required for mating pair formation. Our findings reveal that this physical barrier has two critical consequences for conjugation. First, by preventing plasmid transfer, the capsule acts as a protective shield for the recipient, insulating it from the extensive transcriptional reprogramming and stress responses triggered by plasmid acquisition. We propose that this shielding effect may confer a selective advantage in competitive environments, where maintaining cellular homeostasis is crucial for survival.

Second, we show that the capsule effectively masks the recipient from the donor, preventing the donor from initiating conjugation. Although the necessity of direct cell-to-cell contact for conjugation has been recognized since the 1950s^40^, the role of the recipient during this contact has remained poorly defined. In the case of RP4, stable donor-recipient contact is required for conjugative junction formation^41^, yet previous genetic screens in *E. coli* have failed to identify recipient genes essential for RP4-mediated transfer^42^.

By demonstrating that contact with a capsulated recipient is insufficient to trigger donor activation, we provide compelling evidence that a recipient-encoded envelope factor, obscured by the capsule, is required to initiate donor transcriptional activation. This aligns with recent findings that R388 pilus expression is contact-dependent^43^, but our data suggest that, for RP4, it is the formation of the relaxosome, rather than pilus expression, that is contingent on recipient recognition. Further, we show that contact with capsule is not sufficient to trigger donor activation, showing that the signal for activation is not physical contact alone.

While our findings unequivocally support the prevailing understanding that capsules strongly reduce conjugation efficiency, they also provide a complementary, high-resolution refinement of this model. Earlier population-level measurements of colony volume indicated that the abundance of capsule correlated strongly with conjugation frequency^8^. Here, we show at the single-cell level that capsule thickness precisely determines the physical distance between donor and recipient membranes. This spatial separation is critical, as it governs the likelihood of successful mating pair formation. By examining conjugation with a capsulated recipient at the single cell level, we discovered that rather than functioning as a monolithic barrier that always requires complex genetic events, such as serotype swapping, or a plasmid encoded adaption to be overcome, the capsule is intrinsically “leaky.” Thus, natural variation in capsule thickness at the single-cell and culture level permits occasional RP4 transfer without necessitating genetic adaptation.

Capsular heterogeneity is well-documented across many bacterial species^44–47^, and our findings reveal a novel functional consequence of this variability: it modulates horizontal gene transfer. In *K. pneumoniae*, capsule expression is regulated by complex networks involving over 70 genes^25,48^ and is largely non-phase-variable^25,49,50^. We hypothesise that the observed variation in capsular thickness is predominantly stochastic, as there was no consistent pattern of expression across the population, though this remains to be demonstrated.

While the functional significance of single-cell heterogeneity in conjugation has only recently begun to emerge^51,52^, our study uncovers a new dimension, specifically, that dynamic variation in recipient capsule thickness enables broad-host range plasmids like RP4 to bypass an otherwise effective barrier and disseminate across encapsulated bacterial populations.

In conclusion, we observed that RP4 conjugation was a transcriptionally demanding process for *E. coli* and *K. pneumoniae* recipient cells, extending well beyond the classical SOS response. However, it remains to be determined if this response is specific to RP4 or if other recipient species exhibit distinct responses. Further, we demonstrate that the capsule of *K. pneumoniae* plays a dual role in modulating conjugation: it physically blocks donor-recipient contact, thereby preventing donor activation, and it shields the recipient from the transcriptional stress associated with plasmid acquisition. Finally, through natural, single-cell variation in capsule thickness, RP4 can bypass this defence and successfully transfer into encapsulated populations.

## Materials and methods

### Bacterial strains, plasmids and growth conditions

Table 1 lists the bacterial strains and plasmids used in this work. For routine growth, *E. coli* and *K. pneumoniae* strains were cultured in low-salt LB at 37 °C, shaking in liquid at 200 rpm or on solid media. Media was supplemented with antibiotics where necessary: Ampicillin (100 µg ml^−1^), rifampicin (150 µg ml^−1^), erythromycin (150 µg/ml), spectinomycin (50 µg/ml) and tetracycline (10 µg ml^−1^).

**Table 1:** Bacterial strains and plasmids used in this work

Strain	Genotype/Characteristics	Reference
*E. coli*		
DH5α	F- φ80*lac*ZΔM15Δ(*lac*ZYA-*arg*F)U169 *rec*A1 *end*A1 *hsd*R17(r_K_ m_K_^+^) *pho*A *sup*E44λ^–^*thi*-1 *gyr*A96 *rel*A1	Invitrogen
LT101	Rif^R^ derivative of HB101	^68^
DLL7555	ST73 Rif^R^ clinical isolate of *E. coli*	^69^
*K. pneumoniae*		
B5055	Wild-type *K. pneumoniae* O1:K2	Provided by Richard Strugnell
B5055Δ*wzbc*	Non-encapsulated *wzbc* deletant mutant of B5055, Km^R^	Provided by Richard Strugnell, Adam Jenney, and Tania Uren
SGH10	Hospital isolate of *K. pneumoniae* K1, ST23	^70^
NUH26	Hospital isolate of *K. pneumoniae* K5, ST1049	^71^
NUH40	Hospital isolate of *K. pneumoniae* K5, ST1049	^9^
*A. baumannii*		
AB5075	Wild-type *A. baumanii* KL25, Ap^R^, Erm^S^	^72^
AB10132	Capsular transposon mutant of AB5075, *wzC*::Tn*5*, Tc^R^	^73^
*Pasteurella multocida*		
AL3145	Km^R^ derivative of VPI161	^65^
AL2234	VP161 capsular transposon mutant *hyaD-*Tn*7*, Km^R^	^65^
Plasmids		
pWSK29	Ap^R^, low-copy number cloning vector	^74^
pWSK29 P*traJ*GFPx3	P*traJ*::*mGreenLantern*x3	
pSU18	Cm^R^, medium copy number cloning vector	^75^
pSU18*mTagBFP2*	P*em7*::*mTagBFP2*	This work
pVS520	Tc^R^, Ap^S^, Km^S^, derivative of RP4; Tra^+^ Mob^+^	^69^
pMMB67EH	RSF1010 derived expression vector. Ap^R^, Mob^+^	^76^
RFS1010-*erm*	Erm^R^ derivative of RSF1010, Mob^+^	This work
pAL99S-*oriT*	Spec^R^, Mob^+^ derivative of pAL99S	This work
pMMB67EH*gfpmut3*	P*em7*::*gfpmut3*	This work

*rif* rifampicin, *km* kanamycin, *tc* tetracycline, *ap* ampicillin, *cm* chloramphenicol, *erm* erythromycin, *spec* spectinomycin

### Strain and plasmid construction

Prior to use as recipients, wild-type and Δ*wzbc K. pneumoniae*, along with *E. coli* DLL7555, were electroporated with the low-copy-number vector pWSK29 to provide an ampicillin counter-selection marker for conjugation. Fluorescent reporter plasmids were made as follows. *GFPmut3* or *mTagBFP2* were fused to a synthetic *em7* promoter^53^ and synthesised as gBlocks (IDT). These were cloned into the XbaI and BamHI sites of pMMB67EH or pSU18, respectively, through restriction digestion cloning. pWSK29P*traJ*GFPx3 was similarly constructed, except that three translationally coupled copies of *mGreenLantern* were fused to a 367 bp region upstream of the *traJ* start codon. Each *mGreenLantern* subunit was independently codon optimised until each shared <80% sequence homology.

### Conjugation assays

Overnight liquid cultures of donor and recipient were diluted 1:50 in fresh LB and grown for 4 h before being washed once in PBS and normalized to an OD_600_ of 1.0. 500 µl of donor and recipient culture was passed through a 0.44 µm nitrocellulose membrane filter (Whattman) and incubated on LB agar at 37 °C for 15 min before extraction in 2 ml of PBS by vigorous vortexing. 15 min was chosen as a timepoint to prevent secondary transmission of plasmid RP4 and to minimize growth of the recipient, which can skew transfer frequencies. For routine assays, recipient and transconjugant CFU/ml were quantified by plating onto ampicillin and ampicillin + tetracycline, respectively. For *A. baumannii*, transconjugants were quantified on ampicillin + erythromycin using mobilizable plasmid RSF1010 *erm* as a surrogate for RP4 transfer. For *P. multocida*, kanamycin + spectinomycin using mobilizable plasmid pAL99S-*oriT* as a surrogate for RP4 transfer was performed. Conjugation frequency was calculated as the transconjugant CFU/ml divided by recipient CFU/ml.

### RNA extraction and sequencing

Conjugations were fixed after 15 min of incubation by vortexing mating filters in RNAprotect (Qiagen). Fixed samples were pelleted at 13,300 RPM for 5 min, the RNAprotect was decanted, and samples were stored at -80°C for later processing. RNA was extracted from the DLL7555 samples using an RNeasy RNA extraction kit (Qiagen) as per the manufacturer’s instructions with an on-column DNase treatment (Qiagen). Due to its thick capsule, RNA from *K. pneumoniae* was isolated with hot-phenol as previously described^54^ and further purified using an RNeasy kit with on-column DNase treatment. RNA from DLL7555-related experiments was sequenced at the MHTP Medical Genomics Facility at the Monash Translational Facility. Samples were quality checked by Bioanalyzer (Agilent), and libraries were generated using the Illumina stranded total RNA-seq protocol with ribosomal RNA depletion performed using the bacterial Ribo-Zero rRNA removal kit (Illumina). Sequencing was performed on an Illumina NextSeq550 in high-output mode with chemistry version 2.5, using the Illumina protocol 15046563 version 2. RNA from *K. pneumoniae* related experiments was sequenced at Micromon Genomics. Samples were quality checked by Fragment Analyzer (AATI), and RNA library construction was performed using MGI Tech MGIEasy RNA Directional Library Prep Kit V2 (1000006386). Ribosomal RNA was depleted using the rRNA depletion kit for bacteria (NEB). Resulting libraries were sequenced on an MGIEasy MGISEQ2000RS using V2 chemistry. For all experiments, samples were sequenced to a depth that would ensure at least 10 million reads per isolate in the sample.

### Transcriptomic analysis

Prior to analysis, we assessed the suitability of our two genetically distant *E. coli* donor and recipient strains for RNA-seq analysis. Genetic variation between the donor and recipient was assessed using snippy version 4.6^55^, which detected >75,000 genetic variants, including >68,000 SNPs, which were equally distributed across the recipient genome (Supplementary Fig. 4). This number was in line with previous single-species co-culture RNA-seq experiments^56^. To ensure strain-specific alignment, 53 genes with 100% identity between DLL7555 and HB101 were excluded from the final analysis. RNA reads were aligned against a concatenated sequence of the donor (accession, CP184671.1), recipient (DLL7555, accession CP186931.1 or B5055, assembly GCF_024138975.1), and pVS520 sequences (CP184672.1). The sequence for HB101 (Rif^S^ version of LT101) was used as the donor sequence owing to its availability.

Read alignment was performed with subread version v1.5.1^57^ against the concatenated sequence, which discarded reads of ambiguous donor/recipient origin. >99.8% of donor-originating reads were filtered by subread using this strategy for both B5055 and DLL755 matings, which was assessed by counting the proportion of RNA from a donor-only control that aligned against the recipient portion of the concatenated genome. Read counting and assignment were performed via FeatureCounts^58^. Differential gene expression was analysed with the Voom/Limma method in Degust v4.1 (https://drpowell.github.io/degust/) with a minimum of 3 counts per million across 3 samples to reduce noise from lowly expressed genes, and to exclude genes for which no reads could be uniquely assigned. Replicate consistency was assessed via multidimensional scaling plots within degust using normalised logCPM values for all expressed genes following filtering of lowly-expressed genes (supplementary fig. 5). While biological variation was observed among replicates in the conjugation groups, this was expected given the stochastic and asynchronous nature of active plasmid transfer at 15 min. Importantly, the conjugation and mock conjugation groups demonstrate clear grouping and separation.

Following manual inspection of read alignment within degust, an additional 5 genes (*gatZ*, *fdnG*, *fdnH*, *fdnI* and *gadC*) were subsequently excluded from the DLL7555 analysis because they exhibited donor-specific read counts exceeding 10% of the average recipient read count. Genes were considered differentially expressed if they had a Log2 fold change ≥ 0.585, a *P* value ≤ 0.05, and an FDR ≤ 0.05. This fold change threshold was selected to capture the primary transcriptional response within the short 15-min incubation window while minimizing the detection of transient stochastic noise. GO-term analysis was performed using the TopGO R package v2.58^59^, with the weight_01 algorithm using raw *P* values due to the structured dependency of GO terms as per the package guidelines. eggNOG mapper v2.1.8^60^ was used to assign GO-terms to the GenBank recipient proteomes. Gene and pathway functions were also assessed using the ecocyc database^61^. Heatmaps and GO-term plots were generated using ggplot2 v3.5.2^62^ and the geneviewer package version 0.1.1 (https://github.com/nvelden/geneviewer). Differentially expressed genes that were shared between recipients were determined using OrthoFinder v3.1.2^63^ with default parameters to cluster the recipient DEGS into orthogroups. A shared transcriptional response was defined as any orthogroup containing at least one DEG from both species during active conjugation.

### Statistics and reproducibility

Unless otherwise specified, statistical analyses were performed using GraphPad Prism (v10.0 or later). For quantitative assays such as conjugation frequencies or biofilm assays rank-based tests were primarily employed to ensure robustness. For comparisons between two experimental groups, the two-tailed Mann-Whitney U test was used. For comparisons involving three or more groups, the Kruskal-Wallis test was used, followed by Dunn’s multiple comparison test. Linear regression analysis was used to assess the correlation between capsule thickness and intercellular distance, with the F-test used to determine if the slope was significantly different from zero. Statistical details for RNA-seq analysis are detailed in the transcriptomic analysis section above. Unless specified, experiments were performed with 4 biologically independent replicates.

### Scanning and transmission electron microscopy

SEM was performed on *K. pneumoniae* cells during conjugation, or in stationary phase when capsule is highly expressed^25^. 500 µl of cells were collected onto a 0.4 µm polycarbonate membrane, and the capsular ultrastructure preserved by lysine acetate, ruthenium red and osmium (LRR) fixation^64^, followed by graduated dehydration in ethanol, critical point drying, and gold sputter coating as previously described^65^. Samples were imaged on a field emission NovaSEM 450 SEM with an acceleration voltage of 10 kV in secondary electron mode with a working distance of ~4.5 mm. Images were taken under immersion mode through the lens detector.

For TEM imaging, cells were collected on polycarbonate membranes and prepared with LRR fixation as for SEM. Fixed membranes were then cut into small rectangles no longer than 1 mm cubed, dehydrated and then infiltrated with Epon resin^66^. Each membrane was placed on a glass slide cell-side up, and a BEEM® capsule containing 100% resin was positioned over the flat membrane before polymerisation at 60 °C for 48 h. The resin-filled BEEM® capsule and slide were cooled in liquid nitrogen and then immediately separated, leaving the flat membrane with cells in a longitudinal orientation on the surface of the resin block. Cells were ultrathin sectioned using a Leica UC7 ultramicrotome and Diatome diamond knife and then contrasted with uranyl acetate and lead citrate. Cells were imaged on a JEOL JEM-1400 Plus TEM at 80 KeV, equipped with a high-sensitivity bottom-mount CMOS ‘Flash’ camera. Total capsule diameter was measured from the average of 5 total cell widths from which the cell body width was subtracted, as described previously^67^.

### Fluorescent microscopy and Image analysis

Conjugation was analysed by fluorescent microscopy by inoculation of 1 µl of OD_600_ 0.1 recipient and donor onto LB 1% agarose pads. Pads were incubated in parafilm-sealed 25 mm fluorodish chamber (WPI) for 2 hours at 37 °C. Small strips of moist KimWipe® were included in the chamber to prevent pad dehydration. Images were acquired using a 63x/1.30 oil objective on a Leica DMI-8 widefield microscope equipped with a Lecia K8 sCMOS monochromatic camera using Lecia LasX software. Mating pair productivity was assessed by counting transconjugants that were in direct contact with a donor and dividing that by the total number of recipients in contact with a donor

### Fluorescent reporter assays

Fluorescent measurements were taken on a Tecan Infinite Pro 200 plate reader. For measurement of P*traJ*, 15 µl of *K. pneumoniae* was inoculated onto a 200 µl LB 1% agarose pad with rifampicin in a 96-well special optics microtiter plate (Corning). After 5 min of incubation and air drying, 15 µl of donor culture was spotted on top, and immediately afterwards, the OD600 and fluorescent intensity (488 nm/520 nm) were measured in 5-min intervals at 37 °C. Fluorescent intensity was then normalised to OD600 and analysed as a change over time by calculating fold change against the 0-time point value.

### Reporting summary

Further information on research design is available in the Nature Portfolio Reporting Summary linked to this article.

## Supplementary information

Transparent Peer Review file

Supplementary Information

Description of Additional Supplementary files

Supplementary data 1

Supplementary data 2

Supplementary data 3

Reporting summary

## Data Availability

Transcriptomic data have been submitted to the NCBI SRA under the bioproject accession PRJNA1286074. The data for the numerical values within graphs, differentially expressed genes, and the complete transcriptomic are available within the supplementary material as Supplementary data 1, 2, and 3 respectively. Microscopy data is available upon request.
