# Supplementary material for: Pathogen species and capsule expression shape opportunistic transfer and the recipient response during plasmid RP4 conjugation
Source: Commun Biol. 2026 May 27;9:1127. doi: 10.1038/s42003-026-10359-w (PMC13494036; doi:10.1038/s42003-026-10359-w)
Supplement: Supplementary file 1 [file 42003_2026_10359_MOESM1_ESM.pdf]

nature portfolio

Peer Review File

Pathogen species and capsular expression shape opportunistic transfer and the recipient response during plasmid RP4 conjugation

Corresponding Author: Professor Dena Lyras

This file contains all reviewer reports in order by version, followed by all author rebuttals in order by version.

Version 0:

Reviewer comments:

Reviewer #2

(Remarks to the Author)
The manuscript “Bacterial species and surface structures shape gene transfer and the transcriptional landscape during active conjugation” examines recipient-cell transcriptional responses during RP4 plasmid conjugation and the effect of capsule thickness on transfer efficiency. Using RNA-seq profiling in E. coli and K. pneumoniae, with additional capsule validation in two more species, the study addresses an important question in microbial genetics—how recipient physiology and surface structures influence plasmid acquisition—and provides some data relevant to understanding antimicrobial resistance transfer. The authors’ main claims are that (i) RP4 induces limited and heterogeneous transcriptional responses across species, (ii) the SOS response is not activated, and (iii) thick capsules prevent donor detection and plasmid gene upregulation. While these observations are interesting, the biological conclusions drawn from them remain narrow in scope due to the use of only a single plasmid and a small number of species. Although the authors acknowledge this limitation, it substantially weakens the general impact of the work.

My major concerns are as follows:
1. Only two genes (ompW and nhaA) receive even tentative functional discussion, and these interpretations rely entirely on prior literature. Numerous stress-, metabolic-, and respiration-related genes are highlighted, yet no cohesive interpretation or regulatory framework is developed. As written, the manuscript remains largely descriptive and offers limited mechanistic insight.
2. The exclusive focus on RP4 and a very limited set of host species significantly constrains the applicability of the conclusions. Despite the authors’ caveats, several statements imply broader relevance than the data support.
3. The manuscript reports reduced csgD expression and decreased biofilm formation but cites Ghigo (2001), which demonstrated increased biofilm formation in response to plasmids, including RP4. This discrepancy requires direct explanation, whether due to strain differences, methodological factors, or other biological variables.
4. Greater detail on statistical procedures, replicate consistency, data-filtering criteria, and thresholding decisions is needed to ensure reproducibility and to evaluate the robustness of the analyses.

In summary, while the study raises interesting points about capsule interference and donor–recipient recognition, it lacks sufficient functional depth and mechanistic interpretation. I encourage the authors to expand their functional analyses, incorporate additional plasmids or species, and more clearly develop the mechanistic implications of their findings.

Reviewer #3

(Remarks to the Author)
This manuscript elegantly describes how bacterial conjugation alters the transcription landscape of donor and recipient cells. The authors present compelling data to characterise this complex response and extend their findings to show how capsule production impacts conjugation. The observation of heterogeneity in capsule thickness in the recipient population is exciting and provides a mechanism to explain differences in conjugation efficiency.

The data presented are sound, the manuscript is very well written, and the work addresses an important problem relevant to

increasing antimicrobial resistance.

Overall, I have no substantive concerns regarding the data or major conclusions.

Comments – all minor.

Supplementary Fig 1. Significance stars presented do not match up with the figure legend describing them.

Supplementary Figure 2. Significance values should be added.

There is no reference in the text to Fig 2c. Also, lines 176-187 indicate 10 and 54 DEGs for the wildtype and acapsular K. pneumoniae recipients, respectively. However, in Figure 2c, the Venn diagram of common DEGs add up to more than 10 and 54. I'm not sure if I'm interpreting this incorrectly but it should be checked.

Lines 316-325: Figure 4e should be referred to here.

Version 1:

Reviewer comments:

Reviewer #2

(Remarks to the Author)
There are some valuable observations regarding the comparison of conjugation efficiency between non-capsulated and capsulated individual bacteria. However, the logic used to connect recipient capsulation to the transcriptional cascade in the donor cell is not directly supported at the molecular level. The authors attribute the observed effects primarily to capsule formation, yet no molecular signaling mechanisms—such as ligand–receptor interactions or pilus conformational responses during conjugation—have been identified or experimentally validated to support this link.

While capsulation of the recipient cell may indeed reduce the likelihood of conjugation between two bacterial cells, the manuscript does not provide a sufficiently thorough interpretation of the downstream transcriptional consequences in the donor. In addition, I agree with the first reviewer that the overall scope of the study is rather narrow and limited, and in some instances the conclusions appear to contradict gene functions highlighted in previous literature. For these reasons, it may be premature to draw the broader conclusions currently presented in the manuscript.

Although the authors have addressed many of the questions raised during review, I still have concerns regarding both the nature of the study and aspects of the rebuttal—particularly the opening statement and the response to point no. 2, which appears somewhat superficial.

While the authors have sufficiently addressed points 1, 3, and 4, I continue to have concerns regarding the conceptual framing of the study as well as the way point no. 2 was addressed.

Example:

1. Authors' statement

The authors state:

"We would like to emphasise that a central and unexpected finding of our study is that single cell heterogeneity in capsule expression can be exploited by RP4 to overcome capsule-mediated barriers to conjugation… [text continues] … We believe this single-cell, heterogeneity-driven model represents a substantial conceptual advance in our understanding of conjugative host range expansion and a key contribution of the present work."

We disagree with this interpretation and consider the claim to be both oversimplified and potentially flawed. The authors suggest that their findings "challenge" the prevailing model; however, their results appear instead to largely confirm existing understanding, albeit through a different experimental approach.

It is already well established in the field that capsule thickness strongly inhibits conjugation. Numerous studies demonstrate that conjugation from non-capsulated Escherichia coli donors to various Klebsiella recipients is significantly higher in capsule-deficient mutants compared with capsulated wild-type strains. Importantly, previous work has also shown that the inhibitory effect of capsules is largely independent of their biochemical composition and is instead related to capsule volume and abundance. This framework readily explains fluctuations in conjugation efficiency across strains and may also account for the observations presented in this study.

Furthermore, heterogeneity in plasmid susceptibility to capsule-mediated exclusion is already known. For example, certain plasmids such as pKPN4F can transfer independently of capsule barriers, potentially due to the involvement of interfering alleles such as TraNβ under specific conditions. Thus, the phenomenon of plasmid-specific variation in capsule sensitivity is not entirely novel.

For these reasons, the authors' claim that their data "reveal a previously unrecognised mechanistic pathway by which RP4 circumvents capsule-mediated exclusion without requiring genetic serotype exchange" should be substantially toned down. The evidence presented does not convincingly demonstrate that RP4 actively circumvents capsule-mediated exclusion. Rather, the rare transconjugants observed in the experiments could plausibly be explained by single-cell variability in capsule thickness. Cells with thinner capsules—likely reflecting reduced capsule component levels—would naturally allow occasional conjugation events. This interpretation does not challenge the prevailing understanding that capsules strongly reduce conjugation efficiency.

Point no. 2

The authors state:

"We agree that aspects of the original manuscript… [text continues] … We believe these changes substantially improve precision, prevent over-interpretation, and align the manuscript more closely with the scope and strength of the data."

We believe the revisions made by the authors are not sufficient and that the issue cannot be resolved through minor textual adjustments alone. The limitation in the scope of host species studied remains a key concern. Although the authors have appropriately modified the text to frame the responses as RP4-specific rather than broadly generalizable, this claim may still be questioned.

The limited number of recipient strains examined and the corresponding transcriptional data are insufficient to justify classifying these findings as RP4-specific. We would instead suggest that the authors further narrow their conclusions to the specific strains tested in this study, rather than extending the interpretation to RP4 more broadly. Given that RP4 is one of the most widespread and well-characterized conjugative plasmids, stronger evidence—such as testing additional recipient strains and generating more extensive transcriptional datasets—would be required before making generalized statements about RP4-mediated conjugation.

Version 2:

Reviewer comments:

Reviewer #4

(Remarks to the Author)
I am broadly satisfied with the revised manuscript and I support publication. The study reads as a useful mechanistic explanation of previous work showing that the capsule inhibits conjugation, rather than as a challenge to that model. The main added value is the single-cell and mechanistic resolution of capsule-mediated inhibition during active RP4 conjugation: direct visualization of donor-recipient spacing, evidence that capsule prevents efficient cell-to-cell contact, suppression of RP4 donor gene activation in the presence of capsule, and the link between heterogeneity in capsule thickness and rare successful transfer events.
The transcriptional component is valuable, since the manuscript appropriately restricts its claims to the specific E. coli and K. pneumoniae recipients tested. They show that active RP4 conjugation in these strains is associated with non-SOS stress, respiratory, metabolic, and transport-related reprogramming, and that the capsule can shield K. pneumoniae from this response by reducing conjugation.
A few points of precision would further strengthen the manuscript.
1. The data convincingly show cell-to-cell heterogeneity in apparent capsule thickness and architecture in fixed samples. They do not demonstrate stochastic switching. I wonder if it would be safer to replace "stochastic" with more neutral wording such as "phenotypic heterogeneity in capsule thickness", and softening the statement in the Discussion that "the observed variation in capsular thickness is predominantly stochastic". I still think stochasticity is a plausible and interesting hypothesis, but it remains an hypothesis here.
2. The current wording suggests that earlier population-level approaches are limited in a way that obscures their conclusions. In practice, colony-volume and bulk-capsule measurements are complementary lower-resolution readouts that already established the physical-barrier model and the dependence of conjugation efficiency on capsule serotype and capsule-associated physical properties. The present study adds important spatial and single-cell resolution, and its main findings (that capsule thickness sets donor-recipient spacing, and that thinner-capsule cells account for rare transfer events) are consistent with this prior model. It is indeed fascinating that the authors could show this.
3. I agree with l283: "RP4 can bypass the capsule conjugation barrier through opportunistic transfer into stochastically occurring thin-capsulated recipient sub-populations". I would just remove "stochastically occurring".
4. I agree with l115 "RP4 could opportunistically transfer into genetically thick-capsulated recipients due to single cell variation in capsular thickness, bypassing the need for a serotype switch." Great wording.
Overall, these are very small issues of framing and precision rather than substantive experimental concerns. The manuscript provides a useful mechanistic refinement of capsule-mediated inhibition of conjugation, and I support publication after minor clarification of these points. Congratulations to the authors, I enjoyed reading this manuscript.

Open Access This Peer Review File is licensed under a Creative Commons Attribution 4.0 International License, which permits use, sharing, adaptation, distribution and reproduction in any medium or format, as long as you give appropriate credit to the original author(s) and the source, provide a link to the Creative Commons license, and indicate if changes were made.
In cases where reviewers are anonymous, credit should be given to 'Anonymous Referee' and the source.
The images or other third party material in this Peer Review File are included in the article's Creative Commons license, unless indicated otherwise in a credit line to the material. If material is not included in the article's Creative Commons license and your intended use is not permitted by statutory regulation or exceeds the permitted use, you will need to obtain permission directly from the copyright holder.
To view a copy of this license, visit https://creativecommons.org/licenses/by/4.0/

Point-by-point response to reviewer comments

COMMSBIO-25-10946: **Bacterial species and surface structures shape gene transfer and the transcriptional landscape during active conjugation**

We sincerely thank the reviewers for their thoughtful and constructive evaluation of our work. We have addressed all comments in detail below and revised the manuscript accordingly. The suggested changes have materially strengthened the clarity, interpretation, and overall quality of the manuscript, and we appreciate the opportunity to revise it in response to the feedback.

Reviewer comments are reproduced in full in black text and our responses are in blue text.

Referee 1: Withdrawn

Referee 2:

The manuscript “Bacterial species and surface structures shape gene transfer and the transcriptional landscape during active conjugation” examines recipient-cell transcriptional responses during RP4 plasmid conjugation and the effect of capsule thickness on transfer efficiency. Using RNA-seq profiling in E. coli and K. pneumoniae, with additional capsule validation in two more species, the study addresses an important question in microbial genetics—how recipient physiology and surface structures influence plasmid acquisition—and provides some data relevant to understanding antimicrobial resistance transfer.

The authors’ main claims are that (i) RP4 induces limited and heterogeneous transcriptional responses across species, (ii) the SOS response is not activated, and (iii) thick capsules prevent donor detection and plasmid gene upregulation. While these observations are interesting, the biological conclusions drawn from them remain narrow in scope due to the use of only a single plasmid and a small number of species. Although the authors acknowledge this limitation, it substantially weakens the general impact of the work.

> We would like to emphasise that a central and unexpected finding of our study is that single cell heterogeneity in capsule expression can be exploited by RP4 to overcome capsule-mediated barriers to conjugation. This challenges the prevailing model, as it was thought that capsular serotype switching, *via* recombination with plasmid-encoded capsule loci, was required for a host cell to become conjugation permissive (Haudiquet et al 2024, Manuscript Ref #8).
>
> Using multi-modal microscopy, we demonstrate that capsule expression is heterogeneous at the single-cell level and that this heterogeneity directly governs donor-recipient proximity and conjugation outcomes. Importantly, we further show that capsular heterogeneity coincides with reduced expression of

the capsule biosynthesis locus and its regulators. This, in turn, modulates donor activation through changes in plasmid gene expression, establishing a mechanistic link between recipient surface architecture and donor transcriptional responses.

Together, these data reveal a previously unrecognised, mechanistic pathway by which RP4 circumvents capsule-mediated exclusion without requiring genetic serotype exchange. We believe this single-cell, heterogeneity-driven model represents a substantial conceptual advance in our understanding of conjugative host range expansion and a key contribution of the present work.

My major concerns are as follows:
1. Only two genes (ompW and nhaA) receive even tentative functional discussion, and these interpretations rely entirely on prior literature. Numerous stress-, metabolic-, and respiration-related genes are highlighted, yet no cohesive interpretation or regulatory framework is developed. As written, the manuscript remains largely descriptive and offers limited mechanistic insight.

We thank the reviewer for this thoughtful and constructive critique and agree that the original Discussion did not sufficiently integrate the transcriptional findings into a cohesive biological model. This omission was unintentional and reflected, in part, word count constraints as well as the need to address the capsule-specific components of the study. In response, we have substantially revised the Discussion (Lines 345-383) to present an integrated framework centred on the envelope stress experienced by the recipient during active conjugation, providing clearer biological context for the transcriptional responses observed.

We acknowledge that discussion of individual genes necessarily draws on prior literature; however, we wish to emphasise that this study is, to our knowledge, the first to: A) examine *active* conjugation at this early time point using transcriptome-wide analysis; B) to do so across more than one recipient species; C) directly link transcriptional responses to cell-to-cell contact and conjugation using capsule-deficient mutants; and D) demonstrate that conjugation proceeds at high frequency despite extensive transcriptional perturbation of the recipient. Importantly, while the reviewer's comment is relevant to the transcriptomic component, it does not encompass the detailed experimental and mechanistic analyses of capsule heterogeneity presented here. These data establish how single-cell variation in capsule expression governs donor-recipient interactions, conjugation efficiency, and donor activation, and represent a major and independent advance of the work.

2. The exclusive focus on RP4 and a very limited set of host species significantly constrains the applicability of the conclusions. Despite the authors' caveats, several statements imply broader relevance than the data support.

We agree that aspects of the original manuscript, particularly interpretation of the recipient transcriptional response, could be read as overly generalised. Where relevant, we have explicitly defined these responses as RP4-specific, including clarifying language in the Abstract (e.g., Line 41-42) and introduction

e.g. (Line 115-117). To further ensure appropriate framing, we have revised the title to avoid extrapolation beyond the data presented:

Bacterial species and capsule expression shape gene transfer and the transcriptional landscape during active RP4 conjugation

In addition, we have similarly revised the discussion (Lines 328-344, Lines 440-446) and we have removed the original concluding paragraph (Lines 415-419 in the original manuscript), which implied broader relevance than was supported by the data. The revised concluding paragraph has been rewritten to explicitly limit the interpretation to RP4 and the species examined in this study. We believe these changes substantially improve precision, prevent over-interpretation, and align the manuscript more closely with the scope and strength of the data.

3. The manuscript reports reduced csgD expression and decreased biofilm formation but cites Ghigo (2001), which demonstrated increased biofilm formation in response to plasmids, including RP4. This discrepancy requires direct explanation, whether due to strain differences, methodological factors, or other biological variables.

We thank the reviewer for drawing attention to this discrepancy. We believe it arises from fundamental differences in strain background. The clinical isolate used in our study is highly motile and exhibits strong induction of motility-associated pathways, a response that is likely to delay the transition to a sessile state. In contrast, most laboratory *E. coli* strains, including those used by Ghigo *et al*., are well recognised to be poorly motile or non-motile (Barker *et al*., 2004, manuscript reference #33). We now explicitly discuss this distinction and its implications in the revised Discussion (Lines 368-383).

4. Greater detail on statistical procedures, replicate consistency, data-filtering criteria, and thresholding decisions is needed to ensure reproducibility and to evaluate the robustness of the analyses.

We thank the reviewer for this constructive feedback. In response, we have substantially expanded the transcriptomic analysis section (Lines 512-543) to provide a more detailed description of the bioinformatic pipeline. This includes additional justification for read filtering based on expression level, sequence identity, and removal of contaminating donor reads. We have also clarified our rationale for selecting a Log2 fold-change threshold of 0.585 (1.5-fold). Given the short 15-minute incubation window, chosen specifically to capture primary conjugation events while minimising secondary confounding effects, this threshold balances sensitivity for detecting immediate host responses with sufficient stringency to exclude stochastic transcriptional noise.

To further support data quality and interpretation, we have added a new Supplementary Figure 5 presenting Multidimensional Scaling (MDS) plots for all four RNA-seq datasets. These analyses demonstrate that experimental condition (Mating vs. Mock) is the dominant source of variance, with clear, non-overlapping separation between groups. While some biological variability is observed among conjugation replicates, this is expected given

the stochastic nature of active plasmid transfer during a short experimental window (15 minutes).

We also took this opportunity to strengthen the analysis of conserved transcriptional responses by replacing BLAST-based comparisons with OrthoFinder (Fig 2c). OrthoFinder employs a phylogenetically informed framework to define orthogroups, providing a more robust and interpretable identification of shared responses across species. Importantly, this methodological improvement does not alter the conclusions drawn.

Finally, we have added a dedicated Statistical Procedures section. During this revision, we identified that a parametric ANOVA had been inadvertently applied to Supplementary Figure 3b and have corrected this to an appropriate non-parametric test. This adjustment does not change the statistical interpretation of the data.

In summary, while the study raises interesting points about capsule interference and donor–recipient recognition, it lacks sufficient functional depth and mechanistic interpretation. I encourage the authors to expand their functional analyses, incorporate additional plasmids or species, and more clearly develop the mechanistic implications of their findings.

Our manuscript changes to address these comments are detailed above.

Referee 3:

This manuscript elegantly describes how bacterial conjugation alters the transcription landscape of donor and recipient cells. The authors present compelling data to characterise this complex response and extend their findings to show how capsule production impacts conjugation. The observation of heterogeneity in capsule thickness in the recipient population is exciting and provides a mechanism to explain differences in conjugation efficiency.

The data presented are sound, the manuscript is very well written, and the work addresses an important problem relevant to increasing antimicrobial resistance.

Overall, I have no substantive concerns regarding the data or major conclusions.

Comments – all minor.

Supplementary Fig 1. Significance stars presented do not match up with the figure legend describing them.

We thank the reviewer for identifying this error. The significance symbols have been corrected to match those shown in the figure, and we apologise for the inadvertent mislabelling.

Supplementary Figure 2. Significance values should be added.

We thank the reviewer for this helpful suggestion. Significance values have now been added, and we have corrected an oversight in which significance was incorrectly labelled as $P > 0.05$ rather than ≤ 0.05.

There is no reference in the text to Fig 2c.

We thank the reviewer for drawing our attention to this oversight. The figure has now been correctly referenced at line 200

Also, lines 176-187 indicate 10 and 54 DEGs for the wildtype and acapsular K. pneumoniae recipients, respectively. However, in Figure 2c, the Venn diagram of common DEGs add up to more than 10 and 54. I'm not sure if I'm interpreting this incorrectly but it should be checked.

We agree that the original figure lacked clarity and apologise for the confusion this caused. In response, we have repeated this analysis using a more robust and conceptually appropriate approach, replacing BLAST-based approaches with OrthoFinder. OrthoFinder employs a phylogenetically informed framework to cluster genes into orthogroups, in contrast to BLAST, which relies on pairwise sequence similarity alone. Although this methodological refinement does not materially change the results, it provides a more rigorous identification of conserved responses and is easier to interpret.

In the revised figure, intersections within the Venn diagram now represent shared orthogroups, while the non-overlapping regions indicate the total number of differentially expressed genes within each condition. We have also updated the accompanying table to clearly annotate each shared shared orthogroup, including the corresponding genes and locus tags for each recipient species.

Lines 316-325: Figure 4e should be referred to here.

We have corrected the text to refer to Figure 4e and thank the reviewer for identifying this omission.

Point-by-point response to reviewer comments

COMMSBIO-25-10946A: **Bacterial Species and capsule expression shape gene transfer and the transcriptional landscape during active RP4 conjugation**

We sincerely thank the reviewer for their thoughtful and constructive evaluation of our work. We have addressed all comments in detail below and revised the manuscript accordingly. The suggested changes have materially strengthened the clarity, interpretation, and overall quality of the manuscript, and we appreciate the opportunity to revise it in response to the feedback.

Reviewer comments are reproduced in full in black text and our responses are in blue text. Line numbers refer to the marked-up version of the manuscript.

Response to reviewer:

Reviewer's comment:

The authors state:
"We would like to emphasise that a central and unexpected finding of our study is that single cell heterogeneity in capsule expression can be exploited by RP4 to overcome capsule-mediated barriers to conjugation… [text continues] … We believe this single-cell, heterogeneity-driven model represents a substantial conceptual advance in our understanding of conjugative host range expansion and a key contribution of the present work."

We disagree with this interpretation and consider the claim to be both oversimplified and potentially flawed. The authors suggest that their findings "challenge" the prevailing model; however, their results appear instead to largely confirm existing understanding, albeit through a different experimental approach.

It is already well established in the field that capsule thickness strongly inhibits conjugation. Numerous studies demonstrate that conjugation from non-capsulated Escherichia coli donors to various Klebsiella recipients is significantly higher in capsule-deficient mutants compared with capsulated wild-type strains. Importantly, previous work has also shown that the inhibitory effect of capsules is largely independent of their biochemical composition and is instead related to capsule volume and abundance. This framework readily explains fluctuations in conjugation efficiency across strains and may also account for the observations presented in this study.

Response:

We thank the reviewer for their careful and thoughtful engagement with this point and for highlighting key prior studies that have shaped current understanding of capsule-mediated inhibition of conjugation. We agree entirely that differences between capsule-deficient mutants and capsulated wild-type

strains, as well as between-strain variation across serotypes, are well established determinants of conjugation efficiency. These studies form an important foundation for the present work.

That said, we respectfully suggest that our interpretation differs from existing models in a complementary rather than contradictory way. A key distinction is that previous work has focused almost exclusively on genetically encoded, between-strain or mutant-level differences in capsulation, whereas our study addresses non-genetic, within-strain phenotypic heterogeneity in capsule thickness. To our knowledge, this stochastic single-cell variation within an otherwise genetically uniform population has not previously been directly examined in the context of conjugation, nor incorporated into models of conjugative host range.

With respect to the physical properties of capsule abundance, we acknowledge and cite the recent work by Haudiquet *et al*. (2024), which reported a correlation between colony volume measurements and conjugation frequency. However, this approach does not directly measure capsule thickness or volume at the level of individual cells and is only inferential at colony level. As demonstrated by our TEM and SEM analyses, bulk colony volume is confounded by cell packing density and extracellular void space, which increases markedly in capsulated populations. Thus, such measurements primarily reflect population-scale biomass organisation rather than the physical thickness of capsule associated with individual recipient cells. Similarly, uronic acid-based abundance assays cannot distinguish adherent capsule from shed extracellular material, which we directly visualise in our imaging data. We have clarified these methodological distinctions in the revised Introduction and Discussion (Lines 103-106, 431-445) to better situate our findings relative to prior bulk measurements.

Importantly, bulk assays inherently average across population extremes and are therefore not equipped to resolve stochastic single-cell variation. While existing frameworks successfully predict that genetically “thin-capsulated” strains conjugate more readily than “thick-capsulated” strains, they do not detect or account for the behaviour of phenotypic subpopulations within a single clonal strain. Our central finding is that even a uniformly capsulated genotype contains a dynamic subset of cells with reduced capsule thickness that are permissive for conjugation. These cells provide a plausible and previously unrecognised route by which conjugation can proceed without genetic alteration of the capsule locus.

Finally, by directly quantifying capsule thickness and membrane-to-membrane distances at single-cell resolution, our study provides mechanistic insight into how capsule heterogeneity translates into physical constraints on conjugative contact. We have revised the manuscript to more clearly frame our conclusions as extending and refining, rather than overturning, existing models, and to emphasise that our single-cell observations offer mechanistic resolution that complements prior strain-level and population-level analyses (Lines 431-445).

We appreciate the reviewer's comments, which have helped us clarify the conceptual positioning of the work and better articulate how it builds on, rather than replaces, established understanding.

Reviewer's comment:
Furthermore, heterogeneity in plasmid susceptibility to capsule-mediated exclusion is already known. For example, certain plasmids such as pKPN4F can transfer independently of capsule barriers, potentially due to the involvement of interfering alleles such as TraNβ under specific conditions. Thus, the phenomenon of plasmid-specific variation in capsule sensitivity is not entirely novel.

For these reasons, the authors' claim that their data "reveal a previously unrecognised mechanistic pathway by which RP4 circumvents capsule-mediated exclusion without requiring genetic serotype exchange" should be substantially toned down. The evidence presented does not convincingly demonstrate that RP4 actively circumvents capsule-mediated exclusion. Rather, the rare transconjugants observed in the experiments could plausibly be explained by single-cell variability in capsule thickness. Cells with thinner capsules—likely reflecting reduced capsule component levels—would naturally allow occasional conjugation events. This interpretation does not challenge the prevailing understanding that capsules strongly reduce conjugation efficiency.

Response:

We thank the reviewer for this thoughtful comment and for highlighting prior work demonstrating plasmid-specific variation in sensitivity to capsule-mediated exclusion, including examples such as pKPN4F and the involvement of alleles like TraNβ. We agree that plasmid-encoded adaptations represent an important and well-established mechanism by which certain conjugative elements can bypass capsule barriers under specific conditions. We have no intention of implying that plasmid-specific variation in capsule sensitivity is, in itself, a novel concept.

Our study, however, addresses a mechanistically distinct phenomenon. Rather than proposing that RP4 encodes a specific genetic adaptation that actively counteracts the capsule (as described for TraNβ-containing systems), we show that RP4 transfer can occur opportunistically into a naturally occurring subpopulation of recipient cells that transiently exhibit reduced capsule thickness. In this model, plasmid transfer does not require an active, plasmid-encoded mechanism to overcome the capsule barrier, but instead occurs because phenotypic heterogeneity within an otherwise genetically uniform population creates rare, permissive recipient cells.

We also appreciate the reviewer's observation that our original wording could be interpreted as implying an active circumvention mechanism. We agree that this interpretation would be misleading, and we have therefore revised the manuscript to remove language suggesting that RP4 "actively circumvents" or "overcomes" the capsule barrier. Throughout the revised text, we now use more precise and neutral language that reflects passive, opportunistic transfer into

stochastically thin-capsulated cells (e.g., Lines 40-41, 119-120, 251-253, 267-270, 345-346).

Regarding the reviewer's point that our interpretation does not challenge the prevailing understanding that capsules strongly reduce conjugation efficiency, we fully agree. Our data clearly confirm that capsules impose a profound barrier to conjugation at the population level (Figure 2). Our intention is not to dispute this established principle, but rather to a) visualise how capsule inhibits conjugation, and b) refine how this barrier is conceptualised. Specifically, we suggest that a "thick-capsulated" strain should not be viewed as a uniformly resistant population. Instead, we show that intrinsic single-cell variability in capsule thickness renders this barrier inherently leaky, allowing rare but reproducible conjugation events without the need for genetic serotype exchange or plasmid-specific adaptations.

As the reviewer aptly notes, the rare transconjugants observed could plausibly be explained by single-cell variability in capsule thickness. We agree, and this is precisely the mechanism we propose and directly demonstrate through quantitative single-cell imaging and spatial analysis (Figure 3). By visualising and measuring this heterogeneity, our study provides mechanistic resolution to a phenomenon that has previously been inferred but not directly demonstrated.

We have revised the Discussion to more clearly position our findings as extending and refining existing models, rather than challenging them, and to emphasise that plasmid-encoded adaptations and phenotypic heterogeneity represent complementary, not mutually exclusive, routes by which conjugation can occur in capsulated populations (Lines 431-445). We are grateful to the reviewer for prompting these clarifications, which have improved the precision and framing of the manuscript.

Reviewers comment: Point no. 2

The authors state:

"We agree that aspects of the original manuscript… [text continues] … We believe these changes substantially improve precision, prevent over-interpretation, and align the manuscript more closely with the scope and strength of the data."

We believe the revisions made by the authors are not sufficient and that the issue cannot be resolved through minor textual adjustments alone. The limitation in the scope of host species studied remains a key concern. Although the authors have appropriately modified the text to frame the responses as RP4-specific rather than broadly generalizable, this claim may still be questioned.

The limited number of recipient strains examined and the corresponding transcriptional data are insufficient to justify classifying these findings as RP4-specific. We would instead suggest that the authors further narrow their conclusions to the specific strains tested in this study, rather than extending the interpretation to RP4 more broadly.

Given that RP4 is one of the most widespread and well-characterized conjugative plasmids, stronger evidence—such as testing additional recipient strains and generating more extensive transcriptional datasets—would be required before making generalized statements about RP4-mediated conjugation.

Response:

We thank the reviewer for this careful and substantive critique. We fully agree that, given the limited number of recipient strains examined in this study, our conclusions should not be interpreted as broadly representative of all RP4-recipient interactions. We also agree that more extensive testing across additional recipient species and deeper transcriptional datasets would be required to support more generalised claims about RP4-mediated conjugation. These experiments are important and are currently being pursued as part of future work.

For the present manuscript, however, our aim was not to define a universal transcriptional response to RP4 conjugation, but rather to characterise in detail the recipient responses observed in the specific species examined. We recognise the reviewer's point that even RP4-specific framing may still imply a level of generality that is not fully supported by the current dataset. In response, we have further narrowed the language throughout the manuscript to ensure that all conclusions are explicitly restricted to *Escherichia coli* and *Klebsiella pneumoniae*, the two recipient species directly tested here.

We have comprehensively revised the manuscript to ensure that all statements regarding transcriptional responses are explicitly restricted to *Escherichia coli* and *Klebsiella pneumoniae*, the two recipient species examined in this study. Specifically, the Abstract now limits the analysis to these recipients (lines 32-35), the Introduction defines the scope accordingly (line 74-75), the Results frame all transcriptional analyses as species-specific (lines 127, 171-172), and the Discussion consistently confines interpretation to the species tested while explicitly noting that responses in other recipients or with other plasmids remain unknown (lines 337, 353-354, 360-361, 376,460-463).

We would like to emphasise that it was never our intention to suggest that the transcriptional responses we observe are representative of all RP4 recipients. Rather, our goal was to provide a well-resolved case study demonstrating that active plasmid acquisition can elicit substantial, non-SOS transcriptional responses in specific hosts, and to highlight host- and surface factor-dependent variation in these responses. We believe the revised framing appropriately aligns the scope of our conclusions with the strength of the data, while preserving the central observations of the study.

We are grateful to the reviewer for raising this concern, which has helped us sharpen the framing of the manuscript and avoid overextension of our conclusions.

Point-by-point response to reviewer comments

COMMSBIO-25-10946c: **Bacterial Species and capsule expression shape gene transfer and the transcriptional landscape during active RP4 conjugation**

We are grateful to the reviewer for their time and effort spent evaluating our manuscript. Their thoughtful critique has markedly refined the presentation and strength of the interpretations within our manuscript. We have incorporated all suggested changes, which we have outlined below.

Reviewer comments are reproduced in full in black text and our responses are in blue text. Line numbers refer to the marked-up version of the manuscript.

Response to reviewer:

Reviewer's comment:

Reviewer #4

I am broadly satisfied with the revised manuscript and I support publication. The study reads as a useful mechanistic explanation of previous work showing that the capsule inhibits conjugation, rather than as a challenge to that model. The main added value is the single-cell and mechanistic resolution of capsule-mediated inhibition during active RP4 conjugation: direct visualization of donor-recipient spacing, evidence that capsule prevents efficient cell-to-cell contact, suppression of RP4 donor gene activation in the presence of capsule, and the link between heterogeneity in capsule thickness and rare successful transfer events.

The transcriptional component is valuable, since the manuscript appropriately restricts its claims to the specific E. coli and K. pneumoniae recipients tested. They show that active RP4 conjugation in these strains is associated with non-SOS stress, respiratory, metabolic, and transport-related reprogramming, and that the capsule can shield K. pneumoniae from this response by reducing conjugation.

Response:

We are thankful to the reviewer for their thoughtful and positive assessment of our work. We have addressed the concerns raised below and believe that these changes have markedly improved the clarity, strength and presentation of the findings within our manuscript.

A few points of precision would further strengthen the manuscript.
1. The data convincingly show cell-to-cell heterogeneity in apparent capsule thickness and architecture in fixed samples. They do not demonstrate stochastic

switching. I wonder if it would be safer to replace "stochastic" with more neutral wording such as "phenotypic heterogeneity in capsule thickness", and softening the statement in the Discussion that "the observed variation in capsular thickness is predominantly stochastic". I still think stochasticity is a plausible and interesting hypothesis, but it remains an hypothesis here.

Response:

> We agree with the distinction raised by the reviewer and have removed references to stochasticity in the abstract (L43) and results (L252, L302). Furthermore, we have reframed our discussion of stochasticity as a hypothesis rather than a definitive conclusion (L452-454). This aligns more closely with the presented data, and we thank the reviewer for this thoughtful suggestion.

2. The current wording suggests that earlier population-level approaches are limited in a way that obscures their conclusions. In practice, colony-volume and bulk-capsule measurements are complementary lower-resolution readouts that already established the physical-barrier model and the dependence of conjugation efficiency on capsule serotype and capsule-associated physical properties. The present study adds important spatial and single-cell resolution, and its main findings (that capsule thickness sets donor-recipient spacing, and that thinner-capsule cells account for rare transfer events) are consistent with this prior model. It is indeed fascinating that the authors could show this.

Response:

> We appreciate this perspective and agree that our findings are complementary to prior population-level studies. We apologise for any wording that implied prior conclusions in the field were obscured as this was certainly not our intention. We have reframed our discussion to emphasise how our findings build upon and align with prior studies and the existing physical-barrier model (L426-435).

3. I agree with l283: “RP4 can bypass the capsule conjugation barrier through opportunistic transfer into stochastically occurring thin-capsulated recipient sub-populations”. I would just remove “stochastically occurring”.

Response:

> We agree. We have removed “stochastically occurring” from this sentence, and have ensured consistent terminology throughout the manuscript as indicated in point 1.

4. I agree with l115 “RP4 could opportunistically transfer into genetically thick-capsulated recipients due to single cell variation in capsular thickness, bypassing the need for a serotype switch.” Great wording.

Response:

We sincerely thank the reviewer for this encouraging feedback
