# Supplementary material for: Pathogen species and capsule expression shape opportunistic transfer and the recipient response during plasmid RP4 conjugation
Source: Commun Biol. 2026 May 27;9:1127. doi: 10.1038/s42003-026-10359-w (PMC13494036; doi:10.1038/s42003-026-10359-w)
Supplement: Supplementary file 2 [file 42003_2026_10359_MOESM2_ESM.pdf]

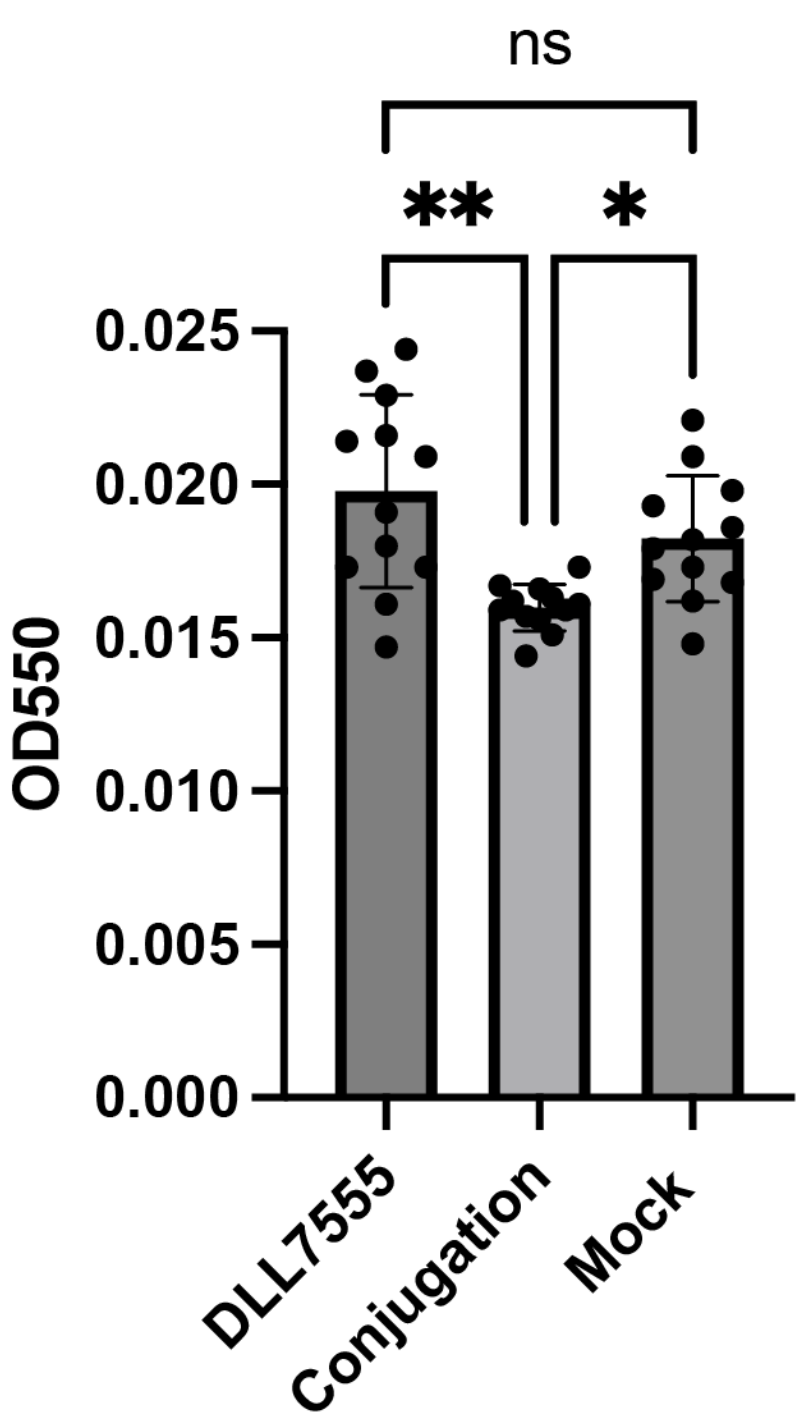

Supplementary Figure 1: Donor LT101(pVS520) and Recipient DLL7555 co-culture causes a minor reduction in biofilm formation.

Biofilms were grown as in (1). Briefly, 1:100 dilutions of overnight broth cultures of donor, recipient and mock donor in fresh LB medium were mixed in 1:1 in a flat bottomed 96 well tray and grown for 24 hours at 37°C. Outer wells were not used to avoid evaporation effects. Following incubation, the liquid portion of the culture was removed and plated to confirm transconjugants were present and therefore conjugation had occurred. The biofilms were washed in water, and quantitated by staining with 0.1% crystal violet, which after washing was solubilized with 30% acetic acid and measured by absorbance reading at 550 nm standardized against 30% acetic acid. Error bars = mean ± SD, N = 4 biological replicates repeated 3 times). Significance testing was performed with a Kruskal Wallis one way ANOVA with Dunn's multiple comparison testing P-values = * ≤ 0.05, ** ≤ 0.01.).

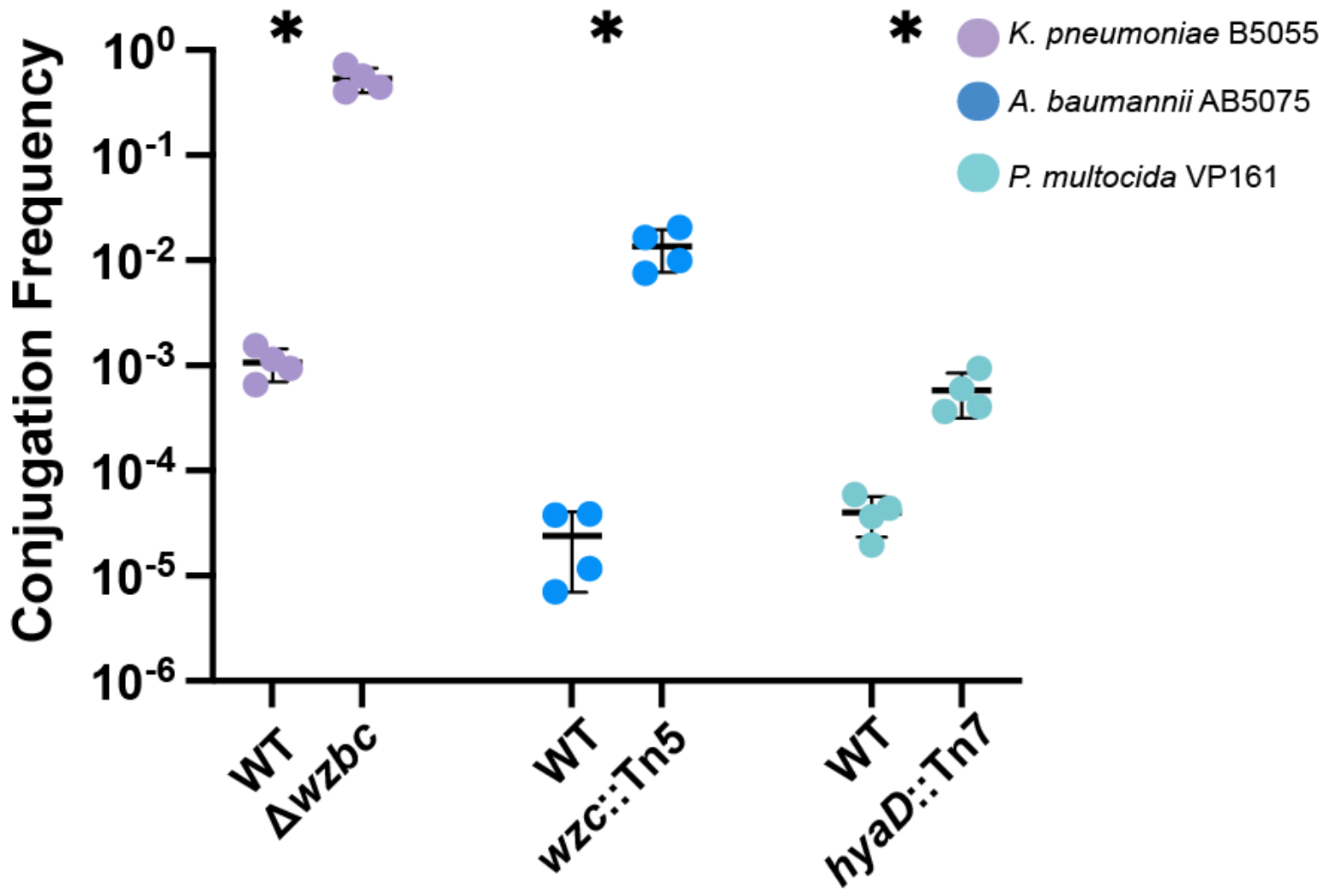

Supplementary Figure 2: Capsule is a barrier to conjugation in diverse species.

The conjugation frequency of RP4 or RP4-mobilizable plasmids from *E. coli* LT101 to K*. pneumoniae* B5055, *A. baumannii* AB5075, or *P. multocida* VP161 and their unencapsulated derivatives (Mean±SD, N=4 Mann-Whitney U test. * = P ≤.05).

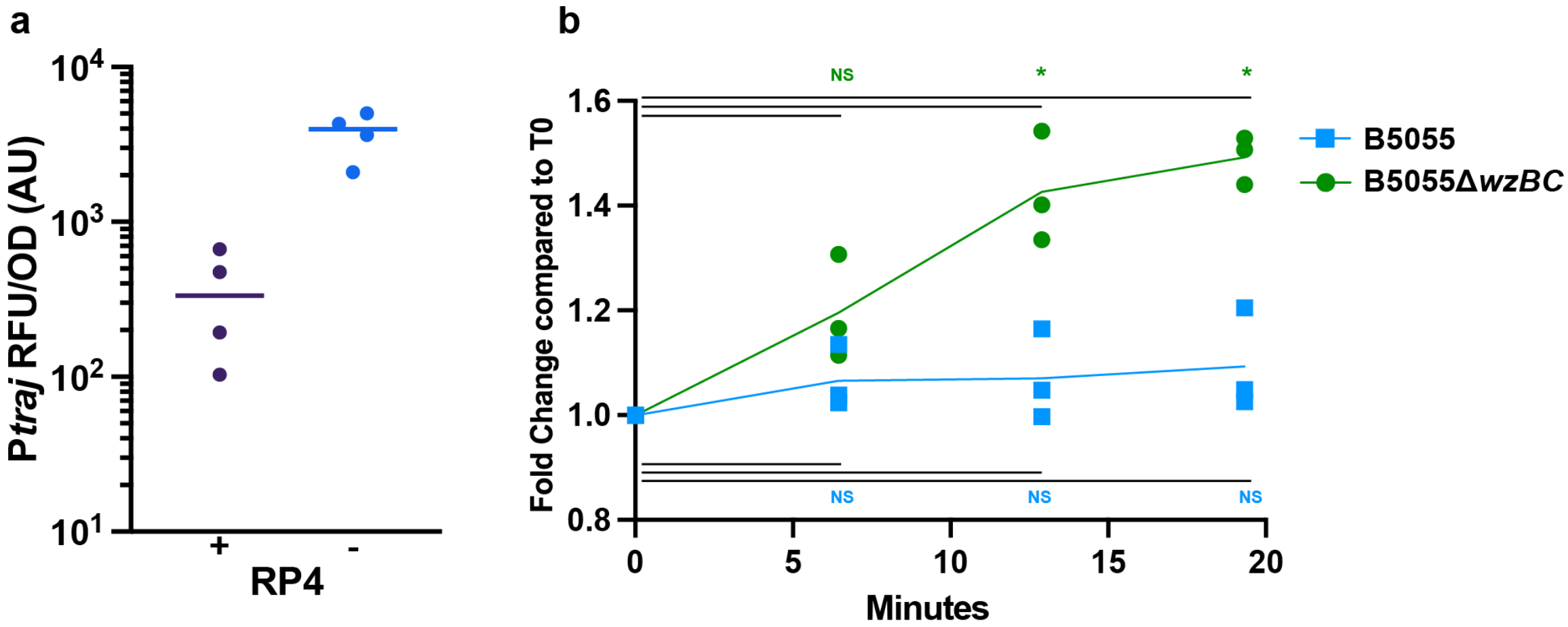

Supplementary Figure 3: Capsule inhibits activation of the RP4 relaxase promoter P*traJ* within the donor. A fluorescent reporter was constructed for the RP4 relaxase promoter P*traJ* by fusing it to three tandem low sequence-homology GFP reporters. Three tandem GFPs were used because fluorescence from 1 GFP reporter could not be detected, likely because P*traJ* is a weak promoter **a)** The presence of RP4 is known to repress P*traJ* and therefore measurement of GFP intensity in the presence or absence of RP4 confirmed the reporter functioned as expected (N = 4, P<0.05, Mann-Whitney U-Test). **b)** Measurement of P*traJ*x3GFP in the donor cell was achieved by performing conjugations on agar containing rifampicin which inhibits transcription only in the recipient cell (N = 3, Kruskal-Wallis one way ANOVA with Dunn's multiple comparison testing, * ≤ 0.05.)

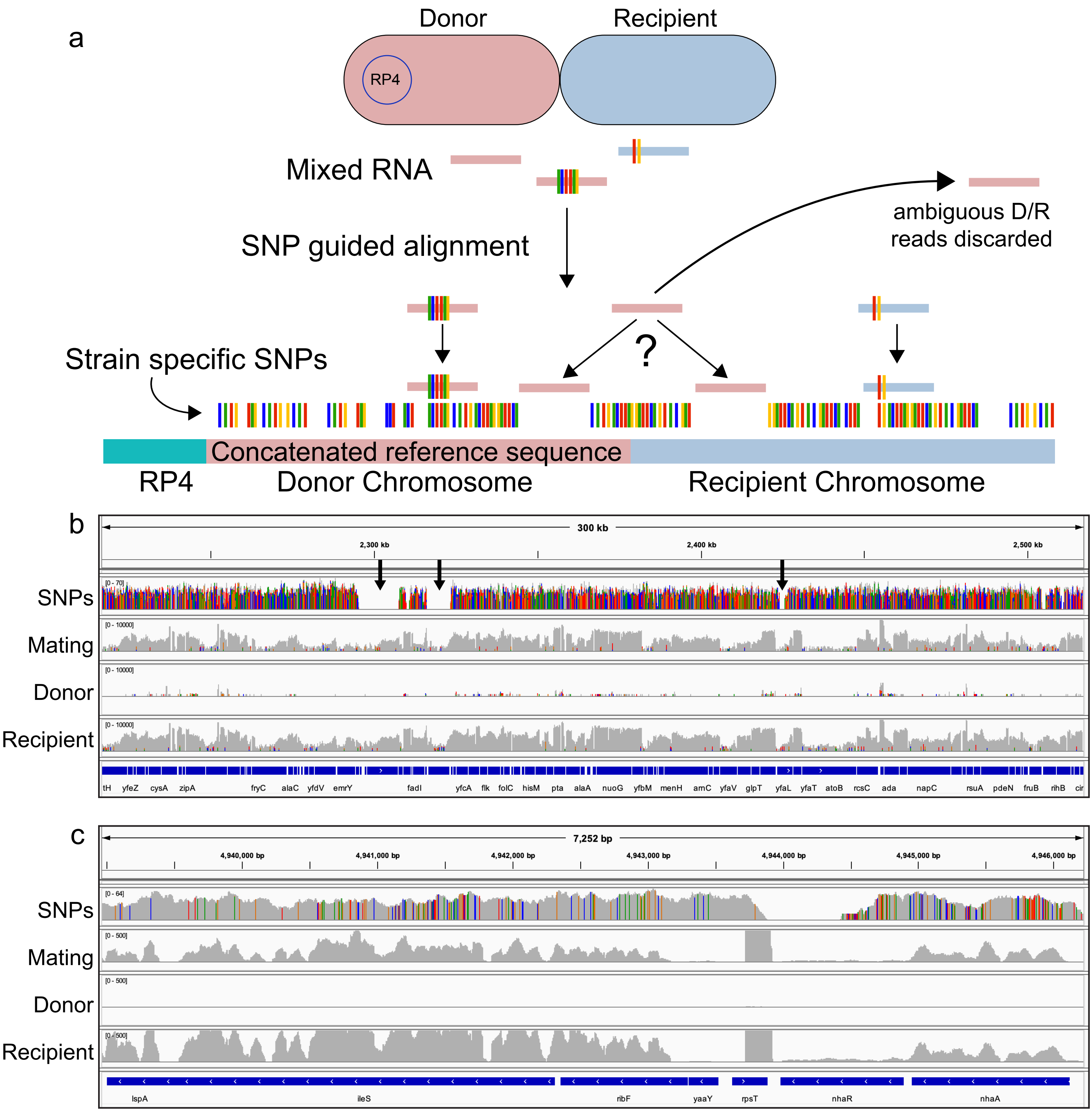

Supplementary Figure 4: SNP and RNA-seq read alignment for HB101(pVS520) x DLL7555. a) Schematic overview of RNA-seq alignment strategy. **b-c)** IGV plots depicting the coverage of SNPs, and the alignment of mating, pure recipient and pure donor read coverage against a recipient portion of the concatenated reference sequence at a global **(b)** and local **(c)** level. Gaps indicated by black arrows in represent genome segments unique to DLL7555. The reads are log scaled in **(b)**.

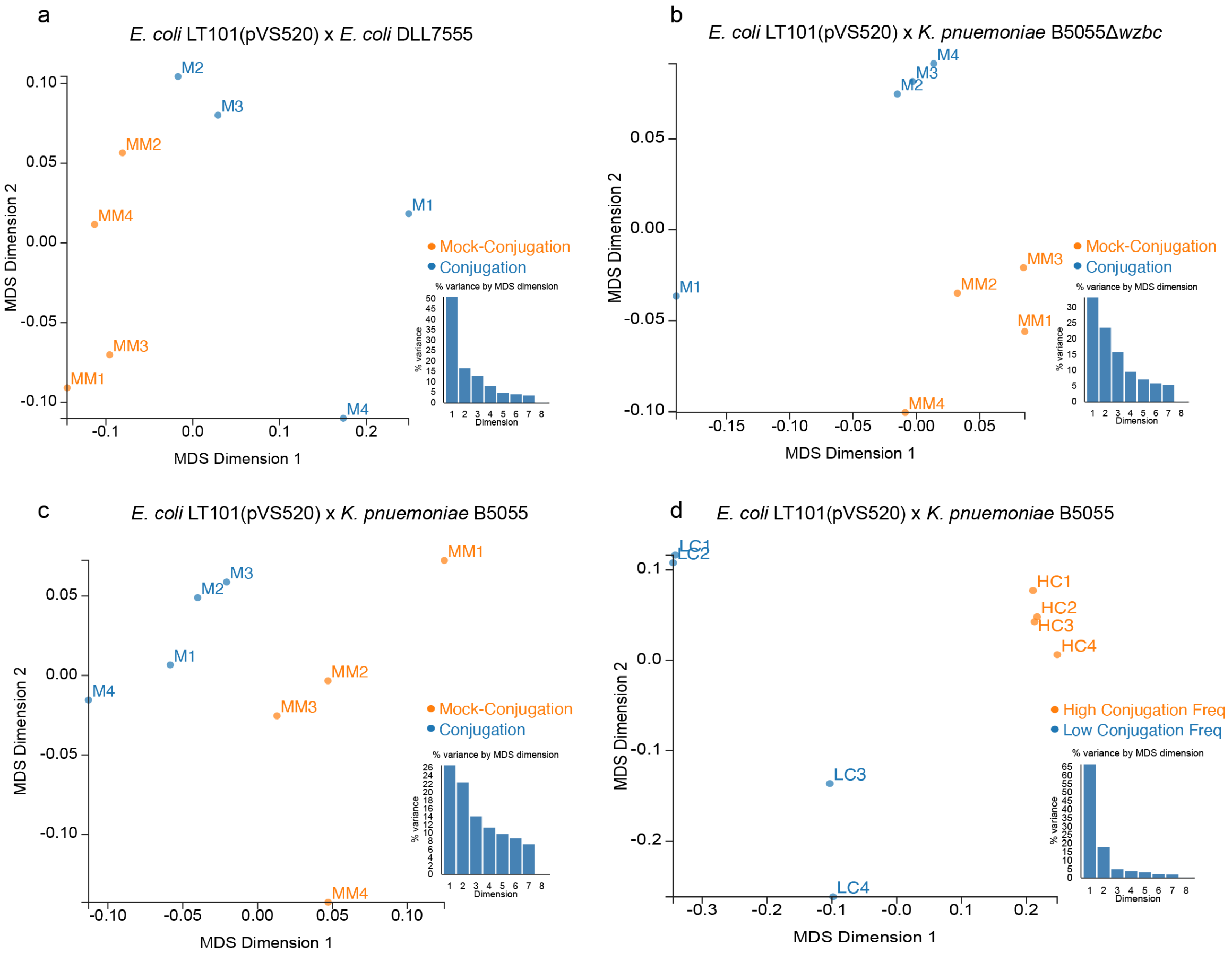

Supplementary Figure 5: Multidimensional Scaling analysis of RNA-seq. MDS plots of global gene expression profiles for the transcriptomic experiments. In each plot, individual points represent biological replicates (N=4 per group), where the distance between points reflects the similarity in their global transcriptomic profiles. Samples are color-coded by experimental condition: orange (Mock-Conjugation or High Frequency) and blue (Active Conjugation or Low Frequency). While biological replicates within the active conjugation groups exhibit some dispersion, this is consistent with the stochastic, and asynchronous nature of conjugation during the 15-minute timepoint.

References

1. B. M. Coffey, G. G. Anderson, Biofilm formation in the 96-well microtiter plate. *Methods Mol Biol* **1149**, 631-641 (2014).
