# Supplementary material for: Pathogen species and capsule expression shape opportunistic transfer and the recipient response during plasmid RP4 conjugation
Source: Commun Biol. 2026 May 27;9:1127. doi: 10.1038/s42003-026-10359-w (PMC13494036; doi:10.1038/s42003-026-10359-w)
Supplement: Supplementary file 3 [file 42003_2026_10359_MOESM3_ESM.pdf]

Description of Additional Supplementary files

File name: Supplementary Data 1

Description: Numerical values for graphs

File name: Supplementary Data 2

Description: Genes called as differentially expressed

File name: Supplementary Data 3

Description: Complete transcriptomic output
